# An αvβ3 integrin checkpoint is critical for efficient T_H_2 cytokine polarisation and potentiating antigen-specific immunity

**DOI:** 10.1038/s41590-022-01378-w

**Published:** 2022-12-22

**Authors:** Aydan C. H. Szeto, Ana C. F. Ferreira, Jonathan Mannion, Paula A. Clark, Meera Sivasubramaniam, Morgan W. D. Heycock, Alastair Crisp, Helen E. Jolin, Patrycja Kozik, Martin D. Knolle, Andrew N. J. McKenzie

**Affiliations:** 1 MRC Laboratory of Molecular Biology, Cambridge, CB2 0QH, United Kingdom; 2 Cambridge University Hospitals, Cambridge, CB2 0QQ, UK

## Abstract

Naïve CD4^+^ T lymphocytes initially undergo antigen-specific activation to promote a broad-spectrum response before adopting bespoke cytokine expression profiles shaped by intercellular microenvironmental cues, resulting in pathogen-focussed modular cytokine responses. Interleukin (IL)-4-induced Gata3 upregulation is important for the T helper 2 (T_H_2) cell polarisation associated with anti-helminth immunity and misdirected allergic inflammation. Whether additional microenvironmental factors participate is unclear. Using whole mouse-genome CRISPR-Cas9 screens we discovered a previously unappreciated role for αvβ3 integrin in T_H_2 cell differentiation. Low-level αvβ3 expression by naïve CD4^+^ T cells contributed to pan-T cell activation by promoting T-T cell clustering and IL-2/CD25/STAT5-signalling. Subsequently, IL-4/Gata3-induced selective upregulation of αvβ3 licenced intercellular αvβ3-Thy1 interactions among T_H_2 cells, enhanced mTOR signalling, supported differentiation and promoted IL-5/IL-13 production. In mice, αvβ3 was required for efficient allergen-driven antigen-specific lung T_H_2 cell responses. Thus, αvβ3-expressing T_H_2 cells form multicellular factories to propagate and amplify T_H_2 responses.

## Introduction

The capacity of CD4^+^ T helper (T_H_) cells to functionally differentiate to provide bespoke responses to specific infections is a critical step in protective host immunity ^1, 2^. Through their specialized production of restricted cytokine repertoires T_H_ cells can adjust immune reactions to particular immune challenges often leading to markedly polarized T_H_ subsets (e.g. interferon-γ (IFN-γ)-producing T_H_1 cells, type-2 cytokine (interleukin-4 (IL-4), IL-5 and IL-13)-secreting T_H_2 cells, IL-17-expressing T_H_17 cells, and IL-21-producing T follicular helper (T_FH_) cells). Multiple diverse signals, including T cell receptor (TCR) signal strength, costimulatory molecules, adhesion molecules, and cytokine milieu, can propagate the transcription factor and epigenetic gene-expression programmes that regulate the specification of these subsets. For example, strong TCR and costimulatory signals lead to IL12Rβ2 upregulation, IL-12 signalling and T_H_1 cell polarization ^3^, whilst low strength T cell activation favours T_H_2 cytokine production ^4^. Furthermore, T cell adhesion via the integrin LFA-1 (αLβ2) favours the development of T_H_1 cells and inhibition of T_H_2 cytokine production ^5, 6, 7^, as well as homotypic T cell aggregation which promotes paracrine IL-2 signalling within T–T clusters ^8^. However, an analogous adhesion molecule-mediated mechanism has not been reported for T_H_2 cell cytokine polarisation during their differentiation.

T_H_2 cells orchestrate immunity to parasitic helminth infections and support tissue repair, but can also drive chronic inflammatory diseases, including allergy and asthma ^2^. During these responses type-2 cytokines induce an immune cascade that involves goblet cell hyperplasia and mucus production, smooth muscle contraction, eosinophilia, mastocytosis, M2-like macrophage polarisation and B cell proliferation and immunoglobulin isotype switching to IgE ^1, 2^. This immune response is set in motion by TCR-mediated T cell activation in an immune microenvironment primed by type-2 initiator cytokines such as IL-25, IL-33 and TSLP, but is highly dependent on instructive signals from IL-4 ligation. IL-4 activates transducer and activation of transcription 6 (STAT6), to induce the canonical T_H_2 cell transcription factor, Gata3, whilst in parallel IL-2 signalling induces STAT5. This combination induces *Il4* transcription and the upregulation of IL-2Ra and IL-4Ra to further potentiate T_H_2 development and cytokine production ^1, 2, 4^. This sets in motion a complex transcriptional and epigenetic network that becomes established during T_H_2 cell differentiation ^2^. However, it is not clear that we have a complete description of fully-validated factors that play roles in T_H_2 cell differentiation, especially cell surface molecules that allow T_H_2 cells to create a microenvironment that promotes efficient polarized cytokine expression and effective type-2 responses. Indeed, although a recent study using an ambitious multiomics and genome-wide CRISPR-Cas9 screening approach reported potential molecular pathways in T_H_2 differentiation, the individual molecules involved remain to be verified functionally in T_H_2 cell differentiation assays and *in vivo* models ^9^. Herein we applied CRISPR-Cas9 whole genome screening to reveal and functionally characterise novel regulators of T_H_2 cell differentiation and function.

## Genome-wide screen for regulators of T_H_2 cells

**Extended Fig. 1:**
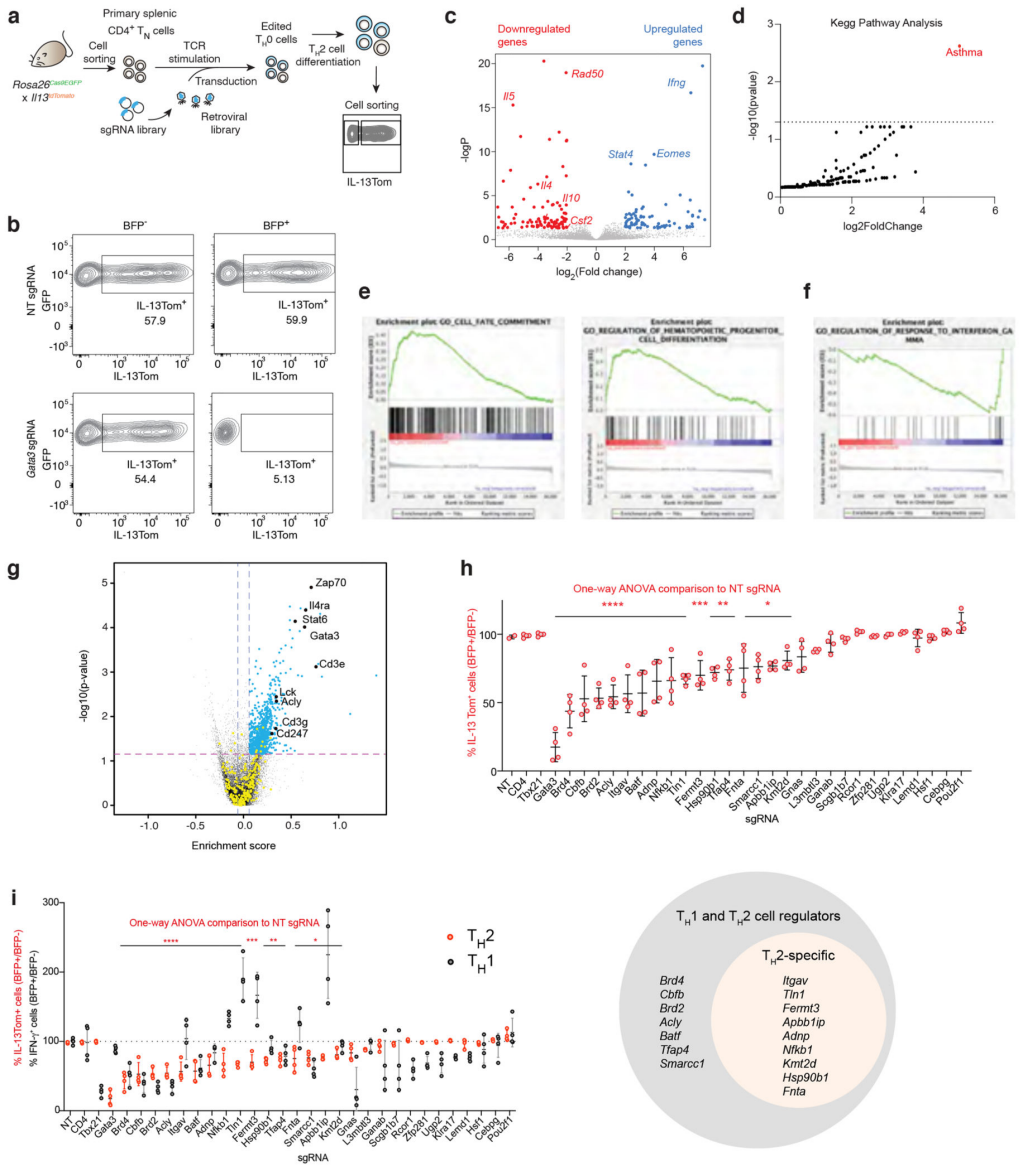
Optimisation of a genome-wide screen for regulators of T_H_2 cell differentiation. (a) Schematic of the optimised T_H_2 cell culture protocol for CRISPR screening. (b) Flow cytometric analysis of IL-13Tom expression by T_H_2 cells transduced with NT or *Gata3* sgRNAs using the optimised protocol. Data representative of 3 independent experiments. (c) RNA-sequencing analysis of *Gata3*-targeted versus non-targeted T_H_2 cells using the optimised screening protocol. (d) KEGG pathway analysis of genes downregulated in *Gata3*-targeted versus non-targeted T_H_2 cells. (e) and (f) Gene set enrichment analyses of genome-wide positive regulators of T_H_2 cell differentiation identified in the screen. (g) Selection of the top 1018 genes from the genome-wide screen for a secondary screen (fold change > 0.06 and p-value < 0.07). (h) Validation of novel regulators by individual confirmatory sgRNA knockdown. Data representative of 3 independent experiments; mean ± SD; one-way ANOVA with Dunnett’s post-hoc test. (i) Validation of novel regulators as in (h) with corresponding T_H_1 comparisons. (h) & (i) **** P<0.0001, ***P=0.0006 (Fermt3), **P=0.0018 (Hsp90b1), 0.0053 (Tfap4), *P=0.0102 (Fnta), 0.0169 (Smarcc1), 0.0212 (Apbb1ip), 0.0252 (Kmt2d).

To identify novel regulators of T_H_2 cell differentiation we optimised an unbiased genome-wide CRISPR screen using primary splenic naïve CD4^+^ T cells from *Rosa26Cas9 x Il13tdTomato* mice (used as a surrogate marker of IL-13 expression and T_H_2 differentiation) (Extended Fig. 1a). To validate the assay, we confirmed that CRISPR-mediated targeting of Gata3 efficiently abrogated IL-13Tom expression in virally transduced (BFP^+^) cells (Extended Fig. 1b) and resulted in the downregulation of 122 genes (4-fold differential expression) including *Il4, Il5, Rad5O and Il10 (Il13* transcripts were not detected due to the use of homozygous *Il13tdTom* alleles), with an enrichment in the asthma-gene-associated pathway (Extended Fig. 1c and d).

**Fig. 1:**
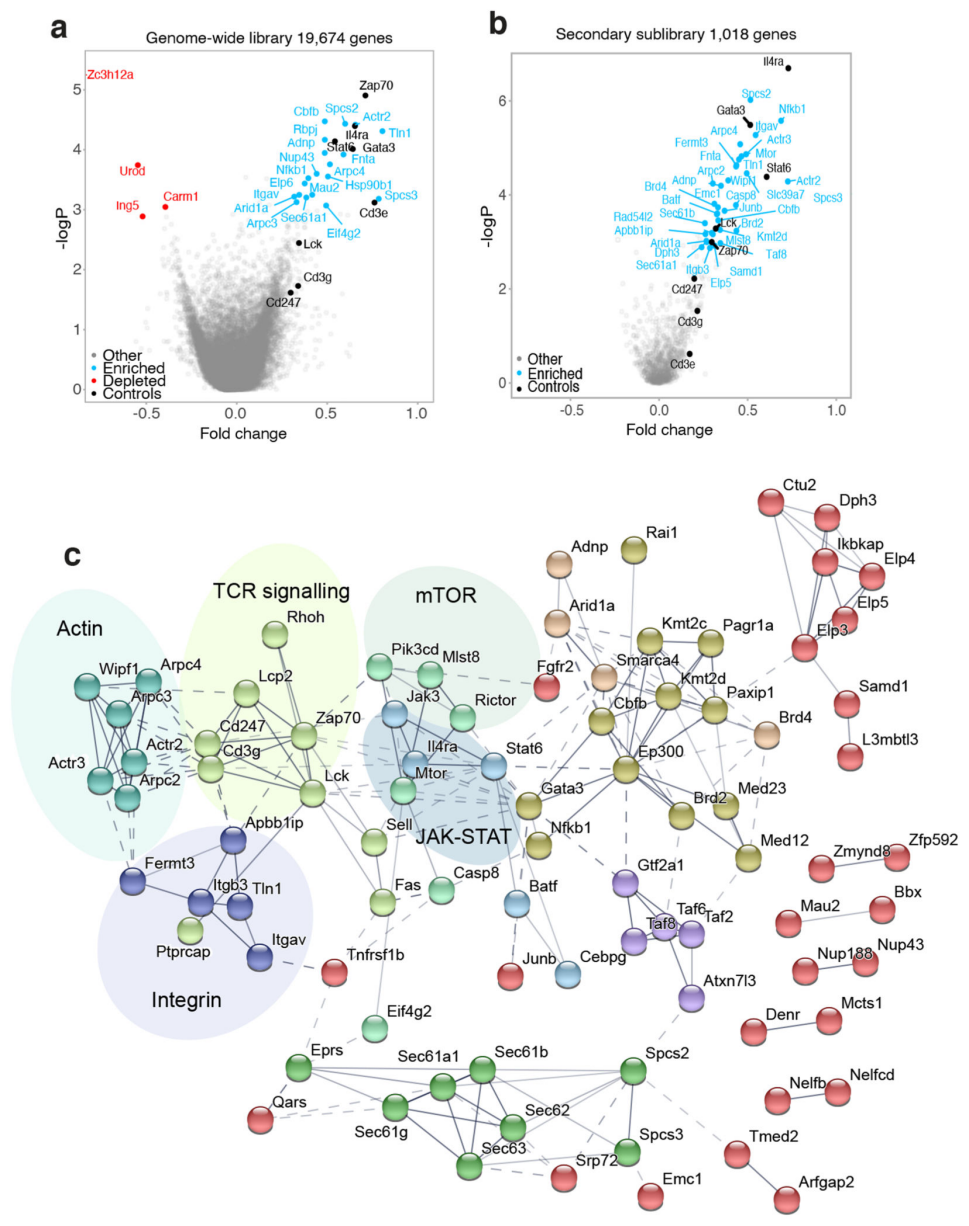
Optimisation of a T_H_2 cell culture protocol compatible with CRISPR screening. (a) Volcano plot showing genome-wide known (black), positive (blue) and negative (red) regulators of T_H_2 cell differentiation, represented as -log(p-value) versus fold change. Data are pooled from 2 independent screens. (b) Volcano plot showing positive regulators of T_H_2 cell differentiation using the secondary sgRNA library, represented as -log(p-value) versus fold change. Data are pooled from 3 independent screens. (c) STRING analysis of the top 100 regulators (top 10%) of T_H_2 cell differentiation identified in the secondary screen. Connecting lines between proteins denote the confidence of the interactions. Kmeans clustering was performed to identify ten clusters represented by individual colours. Co-expression and cooccurrence were removed from active interaction sources. Disconnected nodes (11 proteins) in the network were removed from display.

As anticipated, the genome-wide screens revealed that *Il4ra, Gata3* and *Stat6* were strongly enriched amongst the top positive regulators of T_H_2 differentiation and IL-13 expression ^1, 2^ (Fig. 1a). Other known regulators included *Cd3e, Cd3g, Cd247, Zap70, Rbpj, Nfkb1, Lck, Junb, Batf, Mtor* and *Rictor* (Fig. 1a), while the strongest negative regulator identified, *Zc3h12a*, has been reported recently to negatively regulate T_H_2 cell differentiation *in vivo* ^10^. Gene set enrichment analysis (GSEA) of the top regulators of T_H_2 cell differentiation, ranked according to their sgRNA enrichment in the IL-13Tom^–^ population, revealed an association with the regulation of IL-4 production (FDR q value = 0.021) and T cell receptor signalling (FDR q value = 0.001) (Extended Fig. 1e). In contrast, negative regulators were associated with T_H_1 cell responses, for example *Ifng, Stat1, Ifngr1, Ifngr2* and *Jak2* (Extended Fig. 1f), consistent with a suppressive role of IFN-γ signalling in T_H_2 cell differentiation^11^. Further verification of the top 1018 hits (fold changes > 0.06 and p-values < 0.07) in a secondary pooled screen (Extended Fig. 1g), revealed several novel regulators not previously associated with or fully characterised in T_H_2 differentiation including *Itgav, Acly, Slc39a7, Adnp, Fermt3, Tln1, Apbb1ip, Fnta, Brd2, Brd4, Cbfb* and *Kmt2d* (Fig. 1b).

STRING database analysis of the top 100 genes revealed potential protein interaction modules (Fig. 1c), including TCR signalling pathways, the actin-ARP2/3 module ^12^, mTOR signalling, a JAK-STAT module and chromatin modifiers. Notably, we identified an integrin module ^13^, integrin αv *(Itgav)* and integrin β3 *(Itgb3)*, and the integrin-associated genes *Tln1* (encoding Talin), *Fermt3* (encoding Kindlin-3), *Apbb1ip* (encoding RIAM, RAP1-GTP-interacting adaptor molecule), that associated with T_H_2 cell polarisation and IL-13 expression. Among selected candidates, we performed individual confirmatory knockout assays using 4 sgRNAs per gene which verified 16 candidates as bona fide regulators of T_H_2 cell differentiation (Extended Fig. 1h). Additional analyses of these genes in T_H_1 cell differentiation assays further distinguished T_H_2-specific from shared T_H_1/2 regulators (Extended Fig. 1i). Notably, individual targeting of the integrin-associated genes confirmed their requirement of in T_H_2, but not T_H_1 cell differentiation (Extended Fig. 1h & i).

## Integrin αvβ3 promotes optimal T_H_2 cell responses *in vivo*

**Extended Fig. 2:**
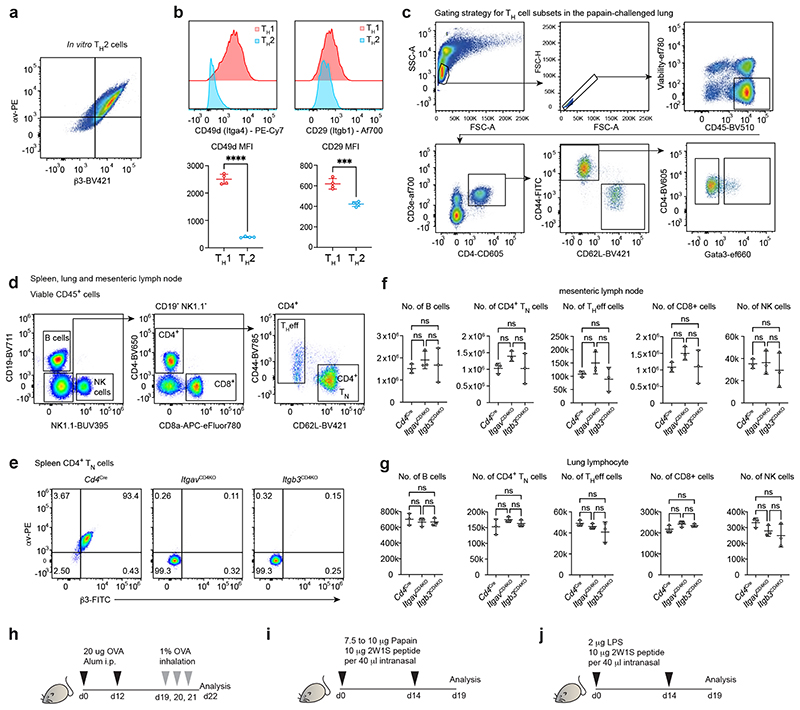
Differential integrin expression by *in vitro* and *in vivo* T_H_ cells (a) Flow cytometric analysis of αv and β3 expression by T_H_2 cells *in vitro*. (b) Flow cytometric analysis of α4 *(Itga4)* and β1 *(Itgbl)* expression by T_H_ cells *in vitro*. Data are representative of 2 independent experiments with 4 biologically independent samples in each experiment; unpaired two-sided t-test; ****P<0.0001, ***P=0.0004. (c) Gating strategy for T_H_ cell subsets in the papain-challenged lung. (d) Flow cytometric gating strategy and quantification of lymphoid populations in naive mice. (e) Flow cytometric analysis of αv and β3 expression in control, αv- or β3-deficient naïve CD4^+^ T cells. (f) and (g) Quantification of lymphoid populations in naive mice. Data representative of 2 independent experiments with 3 biologically independent samples in each experiment; mean ± SD; unpaired two-sided t-test. (h) Schematic of the experimental induction of type 2 inflammation in the mouse lung with OVA/Alum. (i) Schematic of the experimental induction of type 2 inflammation in the mouse lung with papain. (j) Schematic of the experimental induction of type 1 inflammation in the mouse lung with LPS.

**Fig. 2:**
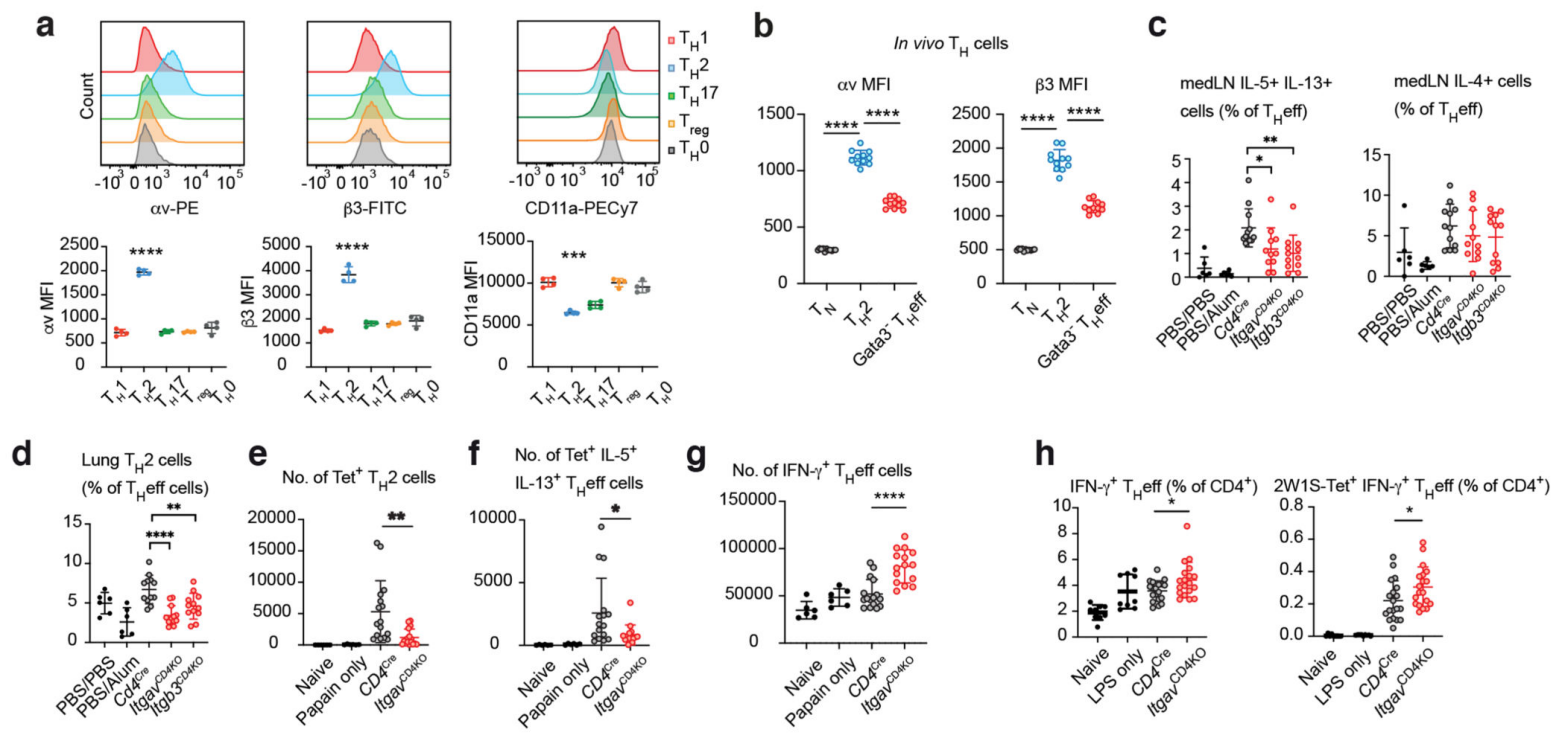
T_H_2 cells differentially express αvβ3 integrin which is required for optimal responses in the lung. (a) Flow cytometric analysis of αv, β3 and CD11a expression by T_H_ cell subsets *in vitro*. Data are representative of 3 independent experiments with n=4 biologically independent samples in each experiment; mean ± SD; one-way ANOVA with Tukey’s post-hoc test; ****P<0.0001, ***P=0.0002 (b) Flow cytometric quantification of αv and β3 MFI in T_H_ cell subsets in the papain-challenged lung. Data pooled from 2 independent experiments representing n=11 biologically independent samples; mean ± SD; one-way ANOVA with Tukey’s post-hoc test; ****P<0.0001. (c) Quantification of type-2 cytokine producing T_H_ effector cells in PMA/ionomycin stimulated mediastinal lymph node cells; **P=0.0059, *P=0.0262. (d) Quantification of T_H_2 cells in lung lymphocytes; ****P<0.0001, **P=0.0047. (e) Quantification of 2W1S-tetramer-specific T_H_2 cells in lung lymphocytes; **P=0.0031. (f) Quantification of 2W1S-tetramer-specific IL-5 IL-13 producing T_H_ effector cells in PMA/ionomycin stimulated lung lymphocytes; *P=0.0260. (g) Quantification of IFN-γ producing T_H_ effector cells in PMA/ionomycin stimulated lung lymphocytes; ****P<0.0001. (h) Quantification of IFN-γ producing T_H_ effector cells in PMA/ionomycin stimulated mediastinal lymph node cells; *P=0.0260 (left) and 0.0443 (right). (c - d) data are pooled from 2 independent experiments and represent mean ± SD (n=6 mice in PBS/PBS and PBS/Alum control groups, n=12 mice in Cd4Cre and Itgb3CD4KO groups, n=11 mice in ItgavCD4KO group); one-way ANOVA with Dunnett’s post-hoc test. (e - g) data are pooled from 2 independent experiments and represent mean ± SD (n=6 mice in naïve and papain only control groups, n=16 mice in Cd4Cre group, n=15 mice in ItgavCD4KO group); unpaired twosided t-test. (h) data are pooled from 3 independent experiments and represent mean ± SD (n=9 mice in naïve and LPS only control groups, n=19 mice in Cd4Cre and ItgavCD4KO groups); unpaired two-sided t-test.

Integrins are heterodimeric cell surface receptors that play critical roles in the cell adhesion and intracellular signalling that contribute to changes in cell morphology, movement, proliferation, survival and differentiation ^14^. We next determined that αv and β3 integrins were most highly expressed on T_H_2 cells compared to other *in vitro* polarised T_H_ cell subsets (Fig. 2a), and that αv and β3 expression was highly correlated (Extended Fig. 2a). This contrasted with the LFA-1 subunit CD11a which was more highly expressed in T_H_1 compared to T_H_2 cells, leading to profound differential expression of T_H_ cell-specific integrin pairs. We also confirmed that integrins CD49d and CD29 were preferentially expressed by T_H_1 cells, as shown previously ^15^ (Extended Fig. 2b). Furthermore, *in vivo*-derived T_H_2 cells expressed higher levels of αv and β3 integrins than non-T_H_2 cells (Fig. 2b & Gating in Extended Fig. 2c).

**Extended Fig. 3:**
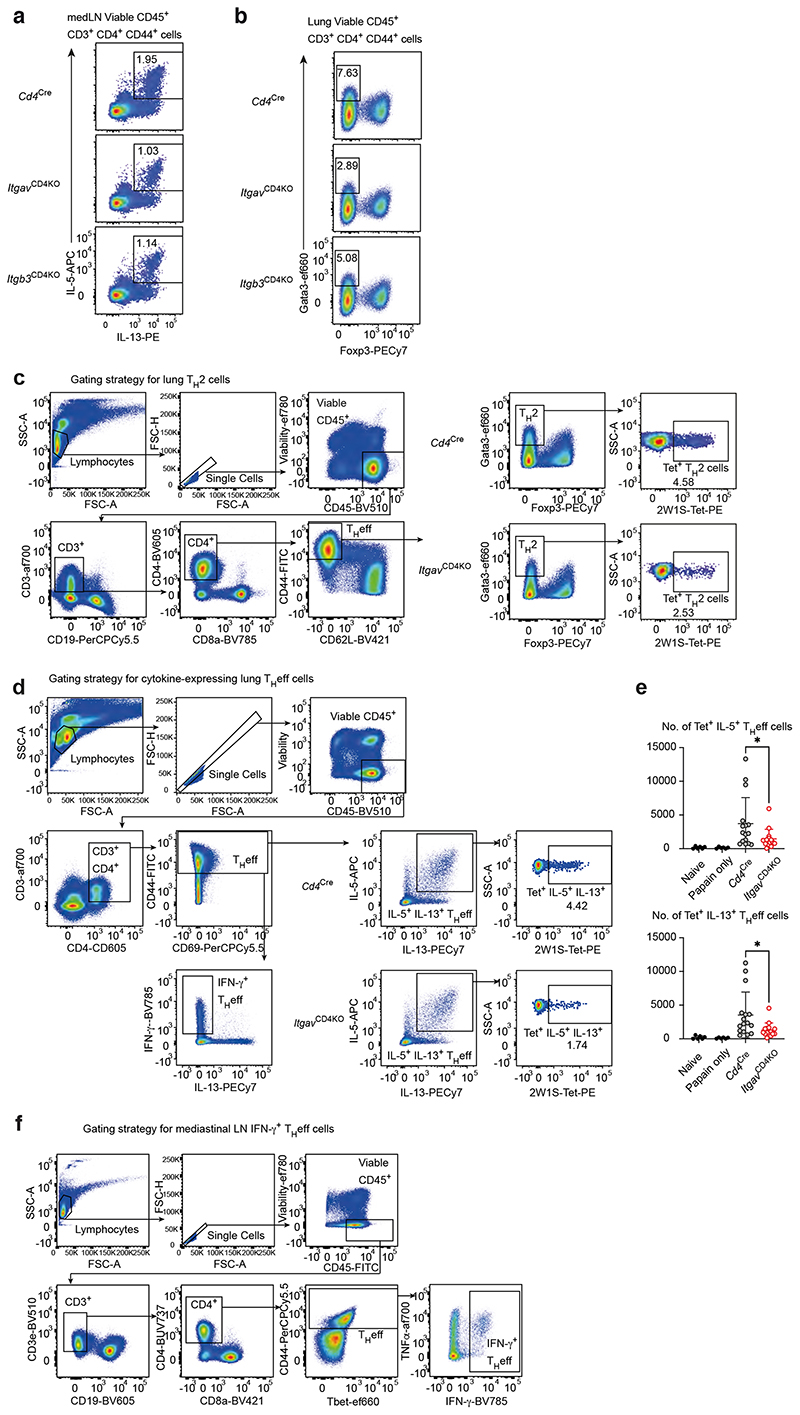
Gating strategy of T_H_ cell populations *in vivo* (a) & (b) Flow cytometric gating strategy for cytokine- and transcription factor-expressing THeff cells in the mediastinal lymph node of OVA/Alum-challenged mice. (c) Flow cytometric gating strategy for 2W1S-tetramer-specific T_H_2 cells in the papain-challenged lung. (d) & (e) Flow cytometric gating and quantification of 2W1S-tetramer-specific IL-5 and IL-13 producing T_H_ effector cells in PMA/ionomycin stimulated lung lymphocytes. Data are pooled from 2 independent experiments and represent mean ± SD (n=6 mice in naïve and papain only control groups, n=16 mice in Cd4Cre group, n=15 mice in ItgavCD4KO group); unpaired twosided t-test; *P=0.0418 (top, IL-5) and 0.0211 (bottom, IL-13). (f) Flow cytometric gating strategy for IFN-γ expressing THeff cells in the mediastinal lymph node of LPS-challenged mice.

To address the potential roles of αvβ3 integrin on T cell immunity *in vivo* we generated conditional αv-deficient *Cd4*^cre^*Itgav*^fl/fl^ (*Itgav*^CD4KO^) and β3-deficient *Cd4*^Cre^*Itgb3*^fl/fl^ (*Itgb3*^CD4KO^) mice, and confirmed αv or β3 ablation in T cells (Extended Fig. 2d and e). Notably, deletion of either αv or β3 abrogated the expression of the other integrin subunit at the cell surface (Extended Fig. 2e). Naïve *Itgav*^CD4KO^ and *Itgb3*^CD4KO^ mice harboured normal frequencies of lymphocyte populations, as compared to *Cd4*^Cre^ controls (Extended Fig. 2f and g). To assess the impact of αvβ3 deficiency on T_H_2 cell responses in the lung we used an ovalbumin/Alum-induced model of pulmonary type-2 inflammation (Extended Fig. 2h). In the absence of αv or β3, a lower proportion of T_H_eff cells produced IL-5 and IL-13 in the lung draining lymph node (Fig. 2c & Gating in Extended Fig. 3a). Interestingly, the T_H_2/T_FH_-associated cytokine IL-4 was not affected (Fig. 2c). Furthermore, a reduction in Gata3^+^ T_H_2 cells was detected in the lungs of *Itgav^CD4KO^* and *Itgb3^CD4KO^* mice (Fig. 2d & Gating in Extended Fig. 3b).

We next employed a lung-allergen challenge model in which the intranasal coadministration of papain and the 2W1S peptide (papain/2W1S) enables the detection of individual endogenous 2W1S antigen-specific CD4^+^ T cells using MHC class II 2W1S-tetramers ^16^ (Extended Fig. 2i). Following allergen priming and challenge we observed a striking impairment in the generation of 2W1S-specific Gata3^+^ T_H_2 cells, as well as antigen-specific IL-5 and IL-13 THeff cells in *Itgav*^CD4KO^ mice as compared to controls (Fig. 2e and f, Gating in Extended Fig. 3c, d and e). Notably, *Itgav*^CD4KO^ mice harboured higher numbers of IFN-γ^+^ THeff cells in the lung (Fig. 2g & Gating in Extended Fig. 3d), further indicating that the T_H_1/T_H_2 cell balance was dysregulated in the context of type-2 inflammation.

Therefore, to assess T_H_1 responses *in vivo* we challenged mice intranasally with bacterial lipopolysaccharide (LPS) in the presence of the 2W1S peptide (LPS/2W1S) (Extended Fig. 2j), which elicited a greater frequency of IFN-γ-producing THeff cells in αv-deficient mice as compared to *Cd4*^Cre^ mice (Fig. 2h & Gating in Extended Fig. 3f). Together, these results demonstrate that the differential expression of αvβ3 on T_H_2 cells is required for efficient differentiation and IL-5 and IL-13 production *in vivo*, and we went on to investigate the underlying mechanism.

## αvβ3 promotes naïve T cell clustering and activation

**Fig. 3:**
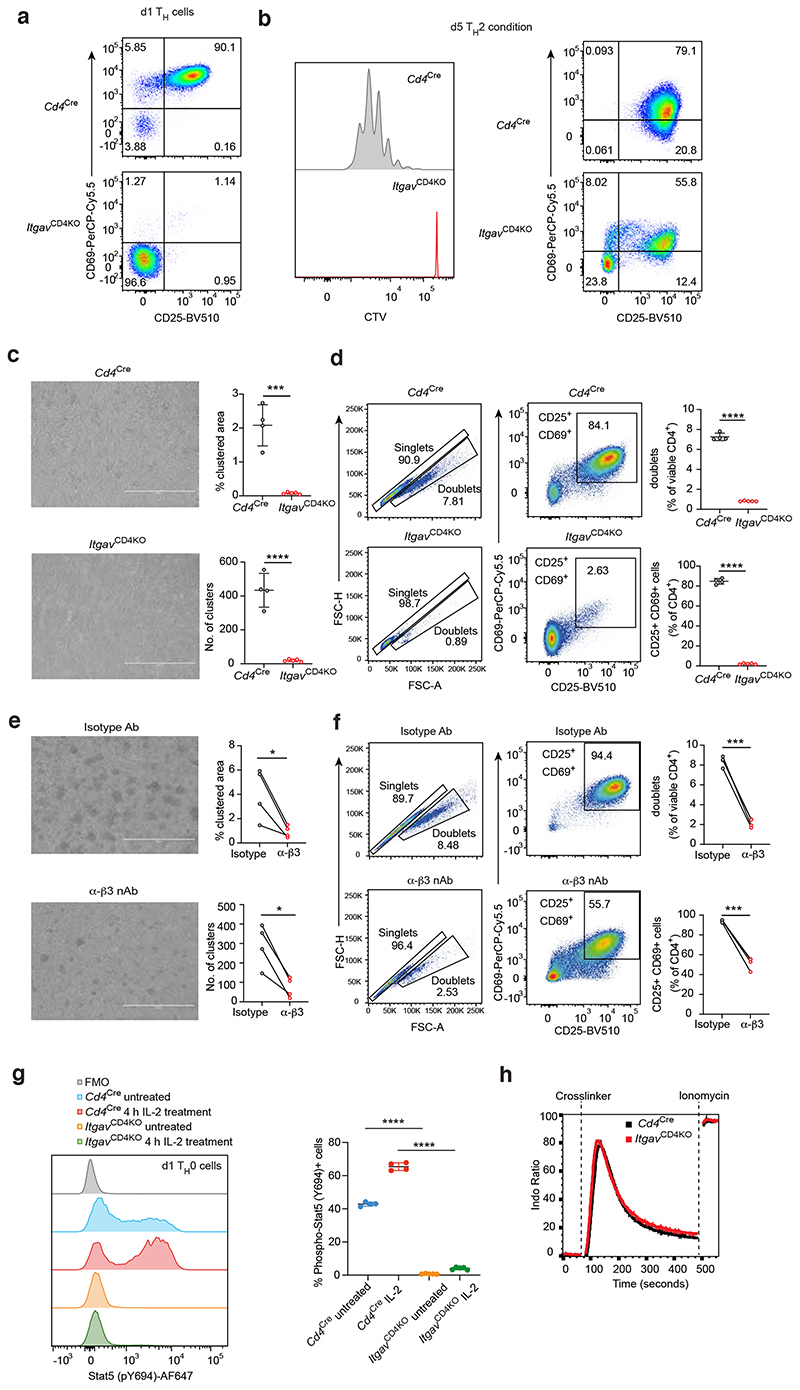
Naïve CD4+ T expression of αvβ3 promotes initial T cell activation and proliferation. (a) Flow cytometric analysis of activation markers by control or αv-deficient T_H_ cells on day 1 post-activation. Data representative of 2 independent experiments. (b) Flow cytometric analysis of activation markers and cell trace dilution of labelled control or αv-deficient T_H_ cells T_H_2 cells. Data representative of 3 independent experiments. (c) Quantification of cell clusters and percentage clustered area of anti-CD3 and anti-CD28 stimulated control or αv-deficient naïve CD4^+^ T cells. Data are representative of 2 independent experiments; mean ± SD; unpaired two-sided t-test; ; ***P=0.0001, ****P<0.0001 (d) Flow cytometric analysis of doublet formation and activation marker expression by T_H_ cells cultured as in (c). Data are representative of 2 independent experiments; paired two-sided t-test; ****P<0.0001. (e) Quantification of cell clusters and percentage clustered area of anti-CD3 and anti-CD28 stimulated naïve CD4^+^ T cells cultured in the presence of isotype or anti-β3 nAb. Data are representative of 2 independent experiments; paired two-sided t-test; *P=0.0361 (top) and 0.0112 (bottom). (f) Flow cytometric analysis of doublet formation and activation marker expression by T_H_ cells cultured as in (e). Data are representative of 2 independent experiments; mean ± SD; unpaired two-sided t-test; ***P=0.0001 (top) and 0.0004 (bottom). (g) Flow cytometric analysis of IL-2-induced Stat5-Y694 phosphorylation in control or αv-deficient d1 T_H_ cells. Data are representative of 2 independent experiments; mean ± SD; unpaired two-sided t-test between the indicated groups; ****P<0.0001 (h) Flow cytometric measurement of TCR-induced calcium flux in control or αv-deficient naïve CD4^+^ T cells. Data are representative of 2 independent experiments with 6 mice per group.

Since integrins such as LFA1 have been shown to play roles in T-T cell conjugate formation during early T cell activation downstream of TCR signalling ^8^ and T_H_1 cell differentiation ^17^, we generated mouse strains to enable us to discriminate possible roles for αvβ3 in the early activation state of T cells (constitutive T cell deletion) and the longer term T_H_2 differentiation process (tamoxifen-inducible T cell-restricted deletion). Indeed, our initial *in vitro* analyses of naïve CD4^+^ T cell cultures from *Itgav*^CD4KO^ mice indicated that αvβ3 was important for the induction of activation markers (CD69 and CD25) (Fig. 3a) and cell proliferation (Fig. 3b), and this correlated with a decrease in T-T conjugate formation as assessed by microscopy (Fig. 3c) and flow cytometry (Fig. 3d). Similar results were obtained using β3 neutralising antibody (Fig. 3e and f).

The deficit in CD25 expression suggested an impairment in IL-2 signalling in the absence of αvβ3. CD25 (IL-2Rα) upregulation and incorporation into the high affinity IL-2Rαβγ complex is required for cell cycle progression and maximal IL-2 responsiveness following CD3 and CD28 stimulation in T_H_ cells ^18^. CD25 upregulation is dependent on IL-2-induced phosphorylation of signal transducer and activator of transcription 5 (STAT5), a key transcription factor in the regulation of CD4^+^ T cell gene transcription, including the transcription of the *Il2ra* gene which results in a loop in which IL-2 promotes T cell proliferation and activation. We tested the importance of this signalling pathway using phospho-flow cytometry to measure IL-2-mediated Stat5 phosphorylation and confirmed reduced Stat5-Y694 phosphorylation in αvβ3-deficient cells (Fig. 3g). To exclude the possibility that αvβ3-deficient cells fail to respond to initial TCR signalling, we confirmed that TCR crosslinking-induced an equivalent calcium flux in control and αv-deficient naïve CD4^+^ T cells (Fig. 3h).

These data revealed that αv integrin-mediated cell adhesion was required to promote the upregulation of CD25 on T cells to potentiate the early events in T cell activation downstream of TCR signalling. Our results indicated that at least *in vitro* αvβ3 performs a previously unappreciated non-redundant role in T_H_ cell activation. However, as our *in vivo* experiments did not reveal any deficit in total THeff cells, it appears that redundant mechanisms exist *in vivo* to compensate for the absence of αvβ3 during pan-T cell activation, for example LFA-1. However, these results did not explain the non-redundant role of αvβ3 in IL-4-induced T_H_2 cell differentiation in our screens and *in vivo* experiments.

## IL-4/Gata3-induced αvβ3 supports T_H_2 cell polarisation

**Fig. 4:**
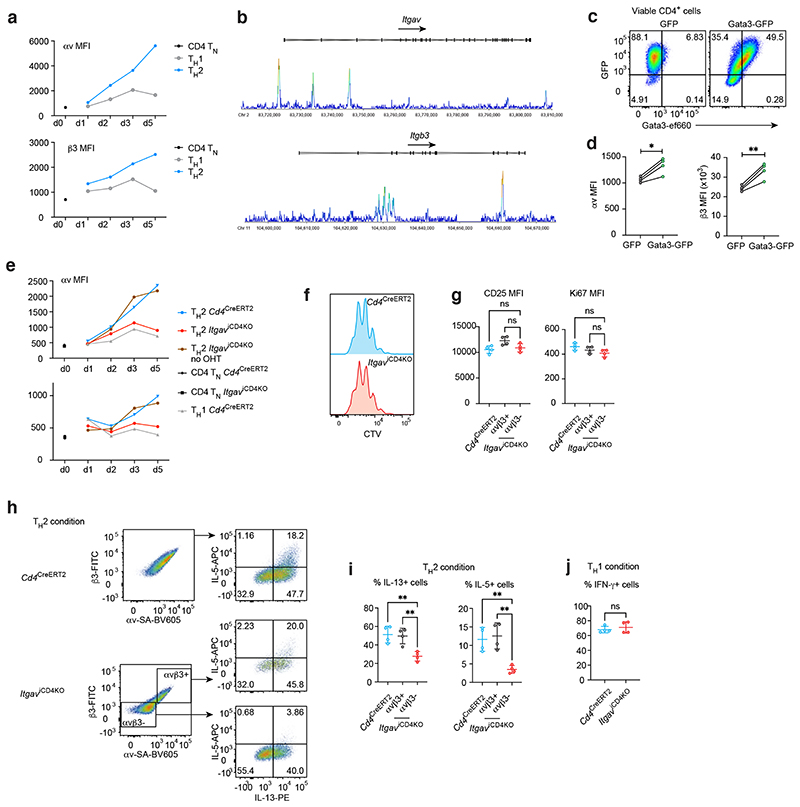
IL-4/Gata3-mediated αvβ3 upregulation is required for T_H_2 cells differentiation *in vitro*. (a) Flow cytometric analysis of αv and β3 expression of naïve CD4^+^ T and differentiating T_H_1 and T_H_2 cells. Data are representative of 2 independent experiments. (b) Gata3 binding to *Itgav* and *Itgb3* loci in T_H_2 cells as assessed by Gata3 ChIP-seq. (c) Flow cytometric confirmation of retroviral Gata3 overexpression and (d) associated analyses of αv and β3 expression in transduced T_H_0 (neutral condition) cells. Data are representative of 2 independent experiments with 4 biologically independent samples in each experiment; mean ± SD; paired two-sided t-test; *P=0.0138, **P=0.0073. (e) Flow cytometric analysis of αv and β3 expression by *Itgav*^iCD4KO^ T_H_ cells cultured with 4-OHT to induce αv or β3 ablation. (f) Flow cytometric analysis of cell trace dilution of labelled T_H_ cells cultured as in (e). (g) Flow cytometric analysis of activation and proliferation markers by T_H_ cells cultured as in (e). Data are representative of 2 independent experiments; one-way ANOVA. (h) - (j) Flow cytometric analysis of cytokine production by T_H_ cells cultured as in (e). Data are representative of 2 independent experiments with 4 biologically independent samples in each experiment; mean ± SD; one-way ANOVA with Tukey’s post-hoc test; **P=0.0050 & 0.0077 (left, IL-13), **P=0.0073 and 0.0037 (right, IL-5).

To interrogate the regulation of αvβ3 expression by IL-4 signalling we performed a time course analysis of αvβ3 expression by T_H_1 and T_H_2 cells. Although αvβ3 is expressed at relatively low levels by naïve CD4^+^ T cells, we observed that IL-4 upregulated αvβ3 expression on T_H_2 cells, while T_H_1 cell-differentiation conditions did not induce αvβ3 (Fig. 4a). IL-4 is an important promoter of T_H_2 cell polarisation through its induction of the transcription factor Gata3, which is essential for type-2 cytokine expression ^19^, and was a prominent hit in our CRISPR screen. By analysing T_H_2 cells using anti-Gata3 chromatin immunoprecipitation-sequencing (ChIP-seq) we identified that Gata3 bound to the promoter of *Itgav* and within the gene body of *Itgb3* (Fig. 4b), indicating the potential of Gata3 to mediate transcription of both integrins. To confirm this, we found that overexpression of Gata3 resulted in the increased cell surface expression of both αv and β3 by T cells (Fig. 4c and d). Together, these results indicated that the IL-4/Gata3 axis can differentially induce αvβ3 on T_H_2 cells as compared to T_H_1 cells.

Thus far our results indicated that αvβ3 expression was upregulated by T_H_2 cells upon IL-4 stimulation (Fig 4b), but was also detectable in lower amounts on naïve CD4^+^ T, T_H_1, T_H_17 and Treg cells (Fig. 2a). Notably, *ex vivo* naïve CD4^+^ T cells express low levels of αvβ3 that were required for early T cell activation whereas high levels of αvβ3 expression, induced by IL-4 and Gata3, were associated with IL-13 and IL-5 expression. This led us to question whether these were distinct pathways which could be separated.

To bypass the requirement for αv integrin in early T cell activation we intercrossed *Itgav*^fl/fl^ mice with *Cd4*^CreERT2^ mice to produce *Itgav*^iCD4KO^ mice in which αvβ3 expression can be temporally deleted from T cells downstream of initial activation. The inducible ablation of αvβ3 was observable from d3 of culture (Fig. 4e), and we observed no defect in T cell proliferation as assessed by CTV dilution and expression of ki67, or the expression of the activation marker CD25 (Fig. 4f and g). Deletion of the integrin subunits was confirmed using flow cytometry with control T_H_2 cells expressing persistently high levels of αv and β3 integrin after tamoxifen treatment, whilst the *Itgav*^iCD4KO^ T_H_2 cell culture contained an increased proportion of αvβ3 negative cells (Fig. 4h). Notably, even in the absence of a proliferative defect the αvβ3-deficient T_H_2 cells expressed reduced amounts of IL-5 and IL-13 compared to both αvβ3-positive T_H_2 cells in the same culture well, and to control T_H_2 cells (Fig. 4i). Although the lower expression of αvβ3 by T_H_1 cells precluded similar in-well analysis to that performed for T_H_2 cells, we observed no difference between control and *Itgav*^iCD4KO^ T_H_1 cells in their expression of IFN-γ (Fig. 4j). These results confirm a role for αv and β3 integrins in T_H_2 cell differentiation that is largely separable from the initial activation/proliferation phenotype. The presence of αvβ3 high T_H_2 cells throughout the culture argues against the involvement of a missing secreted molecule mediating the effects observed in αvβ3-deficient T_H_2 cells.

## αvβ3 promotes FAK-mediated PI3K/mTOR signalling in T_H_2 cells

**Extended Fig. 4:**
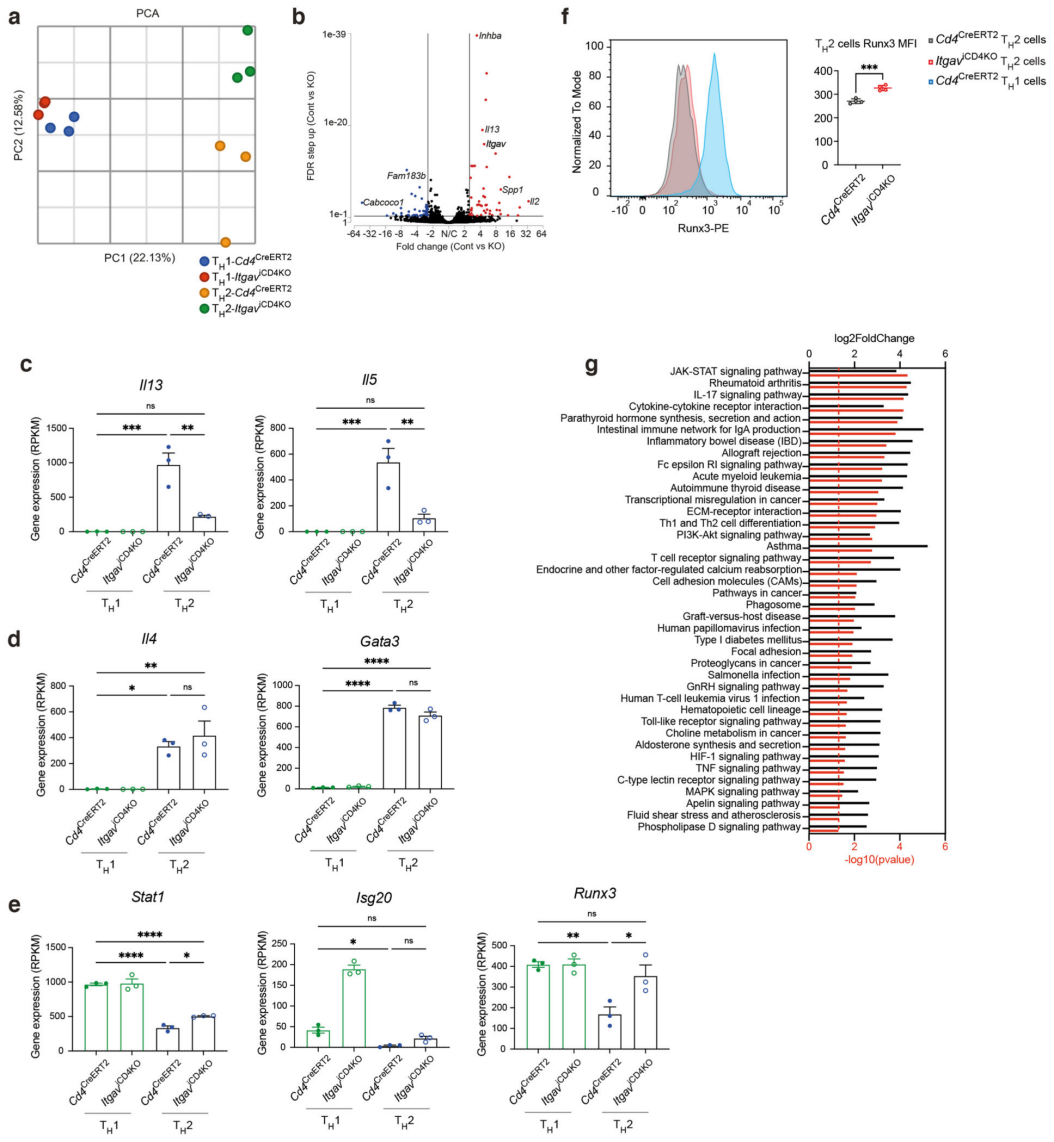
Genome-wide transcriptomic analysis of av-deficient T_H_ cells (a) Principal component analysis of the transcriptomes of control versus av-deficient T_H_ cells. (b) Volcano plot depiction of differentially expressed genes in control versus av-deficient T_H_2 cells. (c) & (d) RNA expression of type 2 genes by control or av-deficient T_H_ cells; *Il13* ***P=0.0002, **P=0.0012; *Il5* ***P=0.0006, **P=0.0025; *Il4* **P=0.0046, *P=0.0167, *Gata3* ****P<0.0001. (e) RNA expression of type-1 signalling genes by control or av-deficient T_H_ cells; *Statl* ****P<0.0001, *P=0.0438; *Isg2O* *P=0.0148; *Runx3* **P=0.0052, *P=0.0220. (c) - (e) mean ± SEM; one-way ANOVA with Tukey’s post-hoc test. (f) Flow cytometric analysis of Runx3 expression by av-deficient T_H_ cells. Data are representative of 2 independent experiments; unpaired two-sided t-test; ***P=0.0003. (g) KEGG pathway analysis of genes downregulated in av-deficient versus control T_H_2 cells.

**Fig. 5:**
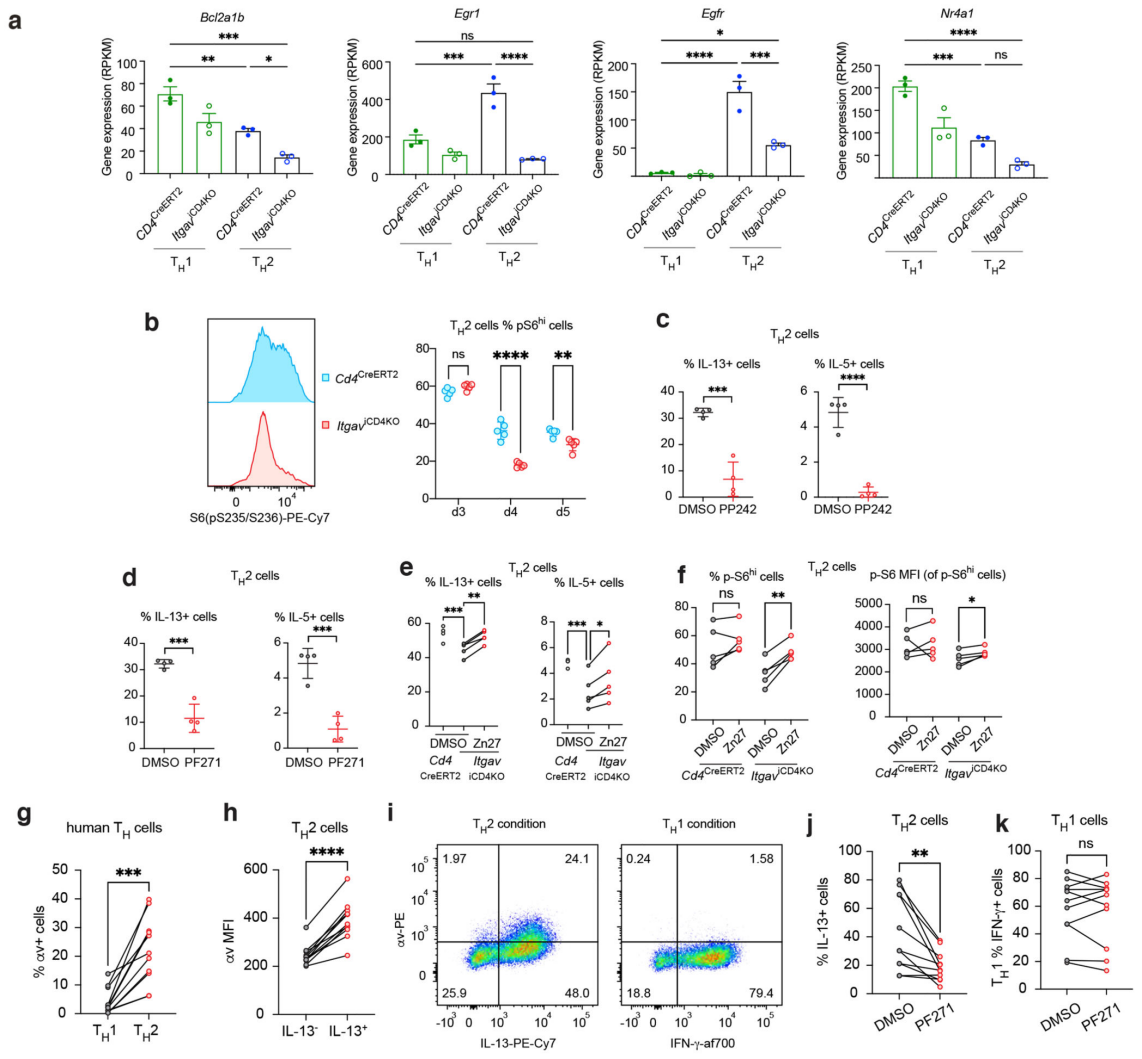
Multiple transcriptomic and signalling perturbations in av-deficient T_H_2 cells revealed by genome-wide transcriptomic analyses and chemical compound treatment. (a) RNA expression of mTOR-regulated genes by control or av-deficient T_H_ cells. Mean ± SEM; one-way ANOVA with Tukey’s post-hoc test; *Bcl21ab* ***P=0.002, **P=0.0074, *P=0.0425; *Egr1* ****P<0.0001, ***P=0.0007; *Egfr* ****P<0.0001, ***P=0.0004, *P=0.0228, *Nr4a1* ****P<0.0001, ***P=0.0007. (b) Flow cytometric analysis of phospho-S6 by control or av-deficient T_H_2 cells. Data are representative of 2 independent experiments; mean ± SD; unpaired two-sided t-test; ****P<0.0001, **P=0.0048. (c) Flow cytometric analysis of cytokine expression by T_H_2 cells cultured in the presence of vehicle (DMSO) or the mTOR inhibitor PP242. Data are representative of 2 independent experiments; mean ± SD; unpaired twosided t-test; ****P<0.0001, ***P=0.0003. (d) Flow cytometric analysis of cytokine expression by T_H_2 cells cultured in the presence of vehicle (DMSO) or the FAK inhibitor PF-271. Data are representative of 2 independent experiments; mean ± SD; unpaired two-sided t-test; ***P=0.0003 (left, IL-13) and 0.0006 (right, IL-5). (e) Flow cytometric analysis of cytokine expression by *Itgav*^iCD4KO^ T_H_2 cells cultured in the presence of vehicle (DMSO) or the FAK activator Zn27. Data are representative of 2 independent experiments; mean ± SD; one-way ANOVA with Dunnett’s post-hoc test; ***P=0.0001, **P=0.0013 (left, IL-13); ***P=0.0001, *P=0.0323 (right, IL-5). (f) Flow cytometric analysis of phospho-S6 by control or av-deficient T_H_2 cells cultured in the presence of vehicle (DMSO) or the FAK activator Zn27. Data are representative of 2 independent experiments; mean ± SD; paired two-sided t-test; *P=0.0146. (g) Flow cytometric analysis of αv expression of human T_H_1 and T_H_2 cells; ***P=0.0002. (h) Flow cytometric analysis of αv expression of human T_H_2 cells; ****P<0.0001. (i) Flow cytometric analysis of αv expression and IL-13 or IFN-γ expression of human T_H_ cells. (j) & (k) Flow cytometric analysis of cytokine expression by human T_H_ cells cultured in the presence of vehicle (DMSO) or the FAK inhibitor PF-271; **P=0.0035. (g) - (k) Data are pooled from 11 volunteers; mean ± SD; paired two-sided t-test.

To further elucidate the mechanism and pathways by which αv deficiency affects T_H_2 cell differentiation, we performed genome-wide transcriptomic analyses on *in vitro* polarised T_H_1 and T_H_2 cells cultured from *Cd4*^CreERT2^ and *Itgav*^iCD4KO^ mice. Principle component analysis (PCA) confirmed the divergence of αv-sufficient and -deficient T_H_2 cells, whereas their T_H_1 cell counterparts were more similar to each other (Extended Fig. 4a). We confirmed that *Itgav*, *Il5* and *Il13* were among the most downregulated transcripts in αv-deleted T_H_2 cells (Extended Fig. 4b and c). The observation that *Il4* expression was unaffected is consistent with previous *in vivo* experiments (Extended Fig. 4d). Furthermore, *Gata3* expression was not dysregulated in αv-deficient T_H_2 cells suggesting that αv is also not required for Gata3 expression (Extended Fig. 4d). Two genes required for IFN-γ signalling, *Stat1* and *Isg20*, as well as the T_H_1-specific transcription factor *Runx3* were upregulated in all αv-deficient T_H_ subsets, likely explaining the T_H_1 bias of T_H_ cell differentiation in αv-deficient cells (Extended Fig. 4e). Increased *Runx3* protein levels in αv-deficient versus control T_H_2 cells were further confirmed using flow cytometry (Extended Fig. 4f). Furthermore, pathway analysis of downregulated genes revealed an enrichment in pathways including asthma, JAK-STAT signalling pathway and PI3K-Akt signalling pathway (Extended Fig. 4g). Consistent with the reduced expression of genes involved in the PI3K-Akt signalling pathway, there were concomitant reductions in genes previously characterised to be regulated by mTOR signalling including *Bcl2a1b*^20^, *Egr1*^21^, Egfr^22^ and *Nr4a1*^23^ (Fig. 5a).

We had also identified an mTOR signalling module (PI3K, Mlst8, mTOR and Rictor) in our CRISPR screens, and we confirmed that αv-deficient T_H_2 cells had reduced mTOR signalling using phospho-S6 as a canonical mTOR signalling readout (Fig. 5b). Furthermore, the mTOR inhibitor PP242 reduced cytokine production from T_H_2 cells (Fig. 5c). To investigate upstream signalling mechanisms that can engage mTOR signalling, we cross-referenced our CRISPR screen results and identified the canonical integrin module including *Tln1* (encoding Talin)*, Fermt3* (encoding Kindlin-3) and *Apbb1ip* (encoding RIAM) with their deletion closely mirroring the *Itgav* deletion phenotype. This suggested the formation of a stereotypical integrin-activation platform. Cell adhesion, mediated by integrins binding to their receptors, also commonly leads to intracellular recruitment and activation of focal adhesion kinase (FAK) and proline rich tyrosine kinase 2 (Pyk2), and mobilisation of the cytoskeleton. In T cells FAK and Pyk2 also lie downstream of T cell receptor activation, and have been proposed to play roles in LFA-1 signalling ^24^. To determine whether inhibition of FAK and/or Pyk2 would phenocopy the αvβ3-dependent effects on cytokine production we employed the FAK/Pyk2 inhibitor PF-562271 (PF271) in T_H_ cell cultures. Treatment of T_H_2 cell differentiation cultures with PF271 impaired IL-13 and IL-5 production as compared to control (Fig. 5d), mirroring the effect of αvβ3 inhibition/deficiency on cytokine production by T_H_2 cells. Next, we assessed whether enforced FAK signalling could rescue the cytokine defect in αvβ3-deficient T_H_2 cells. *Itgav*^iCD4KO^ T_H_2 cells were cultured in the presence of tamoxifen to induce αvβ3 deletion, and additionally with either DMSO (vehicle) or ZINC40099027 (Zn27), an activator of FAK which is reported to interact with the FAK kinase domain and enhance its enzymatic activity for ATP^25, 26^. Notably, culture of αvβ3-deficient T_H_2 cells with Zn27 resulted in an increase in IL-13 and IL-5 production as compared to vehicle controls (Fig. 5e), and reversed the deficit in phospho-S6 in αvβ3-deficient T_H_2 cells (Fig. 5f). Collectively, these results implicate the FAK-mTOR signalling pathway downstream of αvβ3 in T_H_2 cell differentiation and cytokine production.

We extended our analyses to include human T_H_ cells and observed that αv is expressed by a higher proportion of human T_H_2 cells compared to T_H_1 cells (Fig. 5 g). Furthermore, among cells cultured in T_H_2 conditions, αv expression correlated with IL-13 (Fig. 5 h), whereas αv did not correlate with IFN-γ expression among T_H_1 cells (Fig. 5 i). Consistent with data obtained for mouse T_H_2 cells, human T_H_2 cell differentiation was also reduced in the presence of the FAK inhibitor PF271, resulting in a lower proportion of IL-13 expressing cells (Fig. 5 j). In contrast, IFN-γ expression by human T_H_1 cells was unaffected by FAK inhibition (Fig. 5k). These results suggest a similar pathway is utilised by mouse and human T_H_2 cells and warrants further studies on the role of αvβ3-FAK in T_H_2 cell-mediated atopic diseases.

## αvβ3 ligands regulate T_H_ cell differentiation *in vitro*

**Extended Fig. 5:**
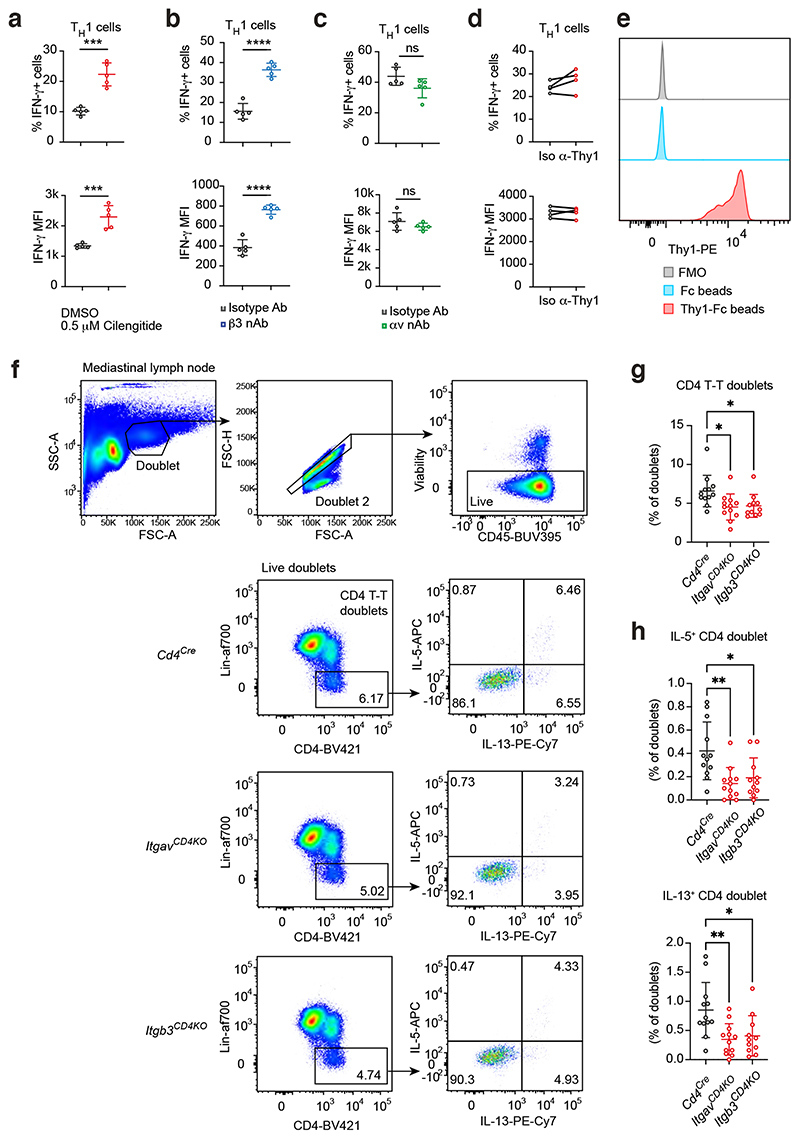
αvβ3-Thyl inhibition does not affect T_H_1 cell differentiation and IFN-γ expression. (a) Flow cytometric analysis of cytokine expression by T_H_1 cells cultured in the presence of vehicle (DMSO) or cilengitide. Data are representative of 3 independent experiments with 5 biologically independent samples in each experiment; mean ± SD; unpaired two-sided t-test; ***P=0.0001 (top, % IFN-γ+ cells) and 0.0005 (bottom, IFN-γ MFI). (b) Flow cytometric analysis of cytokine expression by T_H_1 cells cultured in the presence of isotype or anti-αv antibody. Data are representative of 2 independent experiments with 5 biologically independent samples in each experiment; mean ± SD; unpaired two-sided t-test; ****P<0.0001. (c) Flow cytometric analysis of cytokine expression by T_H_1 cells cultured in the presence of isotype or anti-β3 antibody. Data are representative of 2 independent experiments with 5 biologically independent samples in each experiment; mean ± SD; unpaired two-sided t-test. (d) Flow cytometric analysis of cytokine expression by T_H_1 cells cultured in the presence of isotype or anti-Thyl antibody. Data are representative of 2 independent experiments; paired two-sided t-test. (e) Flow cytometric analysis of Thyl-Fc conjugation to epoxy beads. (f) Flow cytometric gating strategy of CD4 T-T doublets in the mediastinal lymph nodes of OVA/Alum challenged mice. (g) Flow cytometric quantification of CD4 T-T doublets as in (f); *P=0.0132 (Cd4Cre vs ItgavCD4KO), 0.0248 (Cd4Cre vs Itgb3CD4KO). (h) Flow cytometric quantification of type-2 cytokine expressing-CD4 T-T doublets as in (f); IL-5+ CD4 doublet: **P=0.0021, *P=0.0129; IL-13+ CD4 doublet: **P=0.0046, *P=0.0148. (g) – (h) data are pooled from 2 independent experiments and represent mean ± SD (n=12 mice in Cd4Cre and ItgavCD4KO groups, n=11 mice in Itgb3CD4KO group); one-way ANOVA with Dunnett’s post-hoc test.

**Fig. 6:**
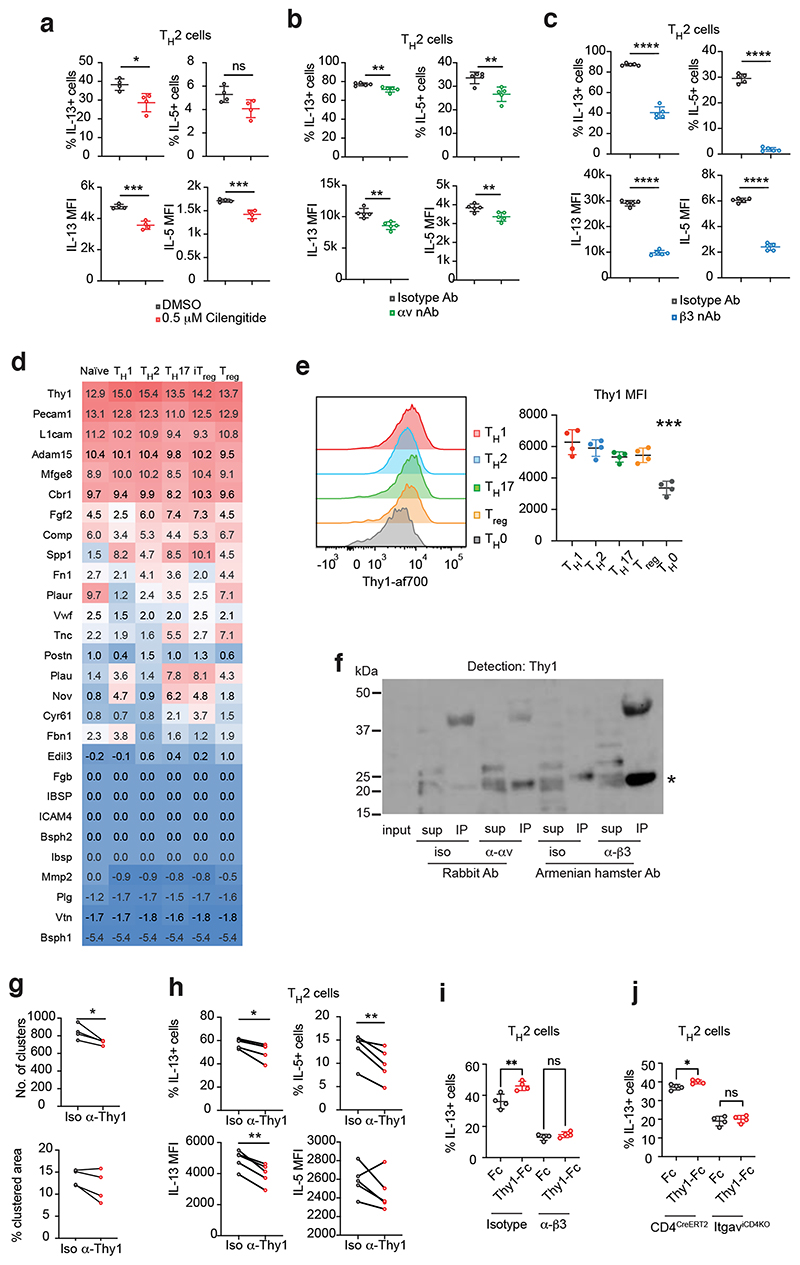
Thy-1-αvβ3 interaction promotes T_H_2 cell differentiation *in vitro*. (a) Flow cytometric analysis of cytokine expression by T_H_2 cells cultured with DMSO or cilengitide. Data are representative of 3 experiments with 4 biologically independent samples in each experiment; mean ± SD; unpaired two-sided t-test; *P=0.0152, ***P=0.0002 (IL-13 MFI) and 0.0010 (IL-5 MFI). (b) Flow cytometric analysis of cytokine expression by T_H_2 cells cultured with isotype or anti-αv antibody; **P=0.0050 (% IL-13+ cells), 0.0043 (% IL-5+ cells), 0.0017 (IL-13 MFI), 0.0089 (IL-5 MFI). (c) Flow cytometric analysis of cytokine expression by T_H_2 cells cultured with isotype or anti-β3 antibody; ****P<0.0001. (b) & (c) Data are representative of 2 experiments with 5 biologically independent samples in each experiment; mean ± SD; unpaired two-sided t-test. (d) Regularised log expression of αvβ3 ligands by T_H_ cells. Data from Th-Express. (e) Flow cytometric analysis of Thy1 expression by T_H_ cells. Data are representative of 2 experiments with 4 biologically independent samples in each experiment; mean ± SD; one-way ANOVA with Tukey’s post-hoc test; ***P=0.0005. (f) Detection of Thy1 protein (25 kDa) in immunocomplexes generated with T_H_2 cell lysate coimmunoprecipitated with anti-αv or anti-β3 antibodies. Data are representative of 2 experiments. (g) Quantification of cell clusters and percentage clustered area of anti-CD3 and anti-CD28 stimulated T_H_ cells cultured with isotype or anti-Thy1 antibody. Data are representative of 2 experiments with 4 biologically independent samples in each experiment; paired two-sided t-test; *P=0.0387. (h) Flow cytometric analysis of cytokine expression by T_H_2 cells cultured in the presence of isotype or anti-Thy1 antibody. Data are representative of 2 experiments with 5 biologically independent samples in each experiment; paired two-sided t-test; *P=0.0145, **P=0.0081 (% IL-5+ cells), 0.0020 (IL-13 MFI). (i) Flow cytometric analysis of cytokine expression by T_H_2 cells cultured in the presence of Fc-of Thy1-Fc-conjugated beads, and additionally with either isotype or anti-β3 antibody; **P=0.0013. (j) Flow cytometric analysis of cytokine expression by *Cd4*^CreERT2^ or *Itgav*^iCD4KO^ T_H_2 cells cultured in the presence of Fc-of Thy1-Fc-conjugated beads; *P=0.0201. (i) – (j) Data are representative of 2 experiments with 4 biologically independent samples in each experiment; unpaired two-sided t-test.

We next addressed the extracellular roles of αvβ3 in T_H_ cell differentiation. The αvβ3 integrin has been reported to bind a variety of extracellular matrix (ECM) ligands containing the arginine-glycine-aspartate (RGD) motif which confers integrin-binding ^27^. We tested the effect of cilengitide, a RGD-containing cyclic peptide which specifically inhibits αvβ3 binding to natural RGD ligands. Cilengitide treatment decreased the percentage of IL-13 expressing cells and the mean fluorescence intensity (MFI) of IL-13 and IL-5 in T cells cultured in T_H_2 differentiation conditions (Fig. 6a). By contrast, inhibiting αvβ3 with cilengitide in THl-polarising cultures resulted in increased proportions of IFN-γ expressing cells and elevated IFN-γ protein expression (Extended Fig. 5a). Furthermore, antibody-mediated neutralisation of αv or β3 decreased IL-13 and IL-5 expression by T_H_2 cells (Fig. 6b and c). Like cilengitide, β3 neutralisation also resulted in an increase in the percentage of IFN-γ expressing T_H_1 cells and IFN-γ production (Extended Fig. 5b), although this effect was not observed when blocking αv (Extended Fig. 5c), possibly due to differential antibody efficacy. These results indicate a T cell-derived extracellular component to the requirement of αvβ3 in T_H_2 cell differentiation (as only T cells were present in the assay).

To identify potential ligands mediating the effects of αvβ3 in T_H_2 cells, gene expression analysis of T_H_ cell subsets identified around 15 candidate ligands for αvβ3 with variable expression (Fig. 6d, Th-express data), with Thyl being the most highly expressed. Thyl (CD90), is a membrane-anchored protein with an extracellular RGD motif that has been demonstrated to bind to αvβ3 and modulate T cell function ^28, 29^. We verified the expression of Thy1 on T_H_2 cells (and other T_H_ cell subsets) (Fig. 6e) and confirmed the interaction of T_H_2 cell-expressed Thy1 with αvβ3 integrins using co-immunoprecipitation assays from cell homogenates with both anti-αv and anti-β3 antibodies (Fig. 6f). Next, we neutralised Thy1 interactions using an antibody which resulted in fewer T-T clusters (Fig. 6g) and reduced IL-13 and IL-5-producing T_H_2 cells in comparison to isotype-antibody treated cells (Fig. 6h), as well as reduced IL-13 MFI (Fig. 6h). By contrast, blocking Thyl did not change the proportion of IFN-γ-producing T_H_1 cells (Extended Fig. 5d). These results support a role for Thy1 in mediating T_H_2 cell clustering and differentiation. It is noteworthy that the impairment of T-T aggregation and cytokine production mediated by blocking Thy1 did not reach those observed when we neutralised the αvβ3 integrin pair. This may indicate that the anti-Thy1 blocking antibody is less efficient than those used for inhibiting the integrins, or that additional semi-redundant αvβ3 ligands exist amongst the potential ligands expressed by T_H_2 cells. Such redundancy of the ligands is also suggested by the whole genome CRISPR-screen failing to identify a potential ligand for the αvβ3 integrin pair.

To confirm a role for Thy1 we cultured T_H_2 cells at low density to reduce cell-cell contact but provided exogenous Thy1 ligand in the form of recombinant Thy1-Fc conjugated to beads (Extended Fig. 5e). We found that Thy1-Fc-conjugated beads increased the percentage of IL-13-expressing cells in T_H_2 cell culture, compared to Fc-conjugated beads (Fig. 6i). This enhancement was abrogated when β3 was neutralised (Fig. 6i), and similar results were obtained using αv-deficient Itgav^iCD4KO^ T_H_2 cells (Fig. 6j). These results validate Thy1 as a physiological αvβ3 ligand and highlight the role of Thy1-αvβ3 interactions in promoting T-T interactions and T_H_2 cell differentiation.

To corroborate the role of αvβ3 in mediating T-T cluster formation *in vivo*, we analysed T-T doublets in mice challenged with OVA/Alum, a protocol that generates robust T_H_2 cell responses in the mediastinal lymph node (Extended Fig. 5f). αv- and β3-deficient mice harboured lower proportions of CD4 T-T doublets (Extended Fig. 5g) and IL-5/IL-13 expressing CD4 T-T doublets (Extended Fig. 5h) compared to control mice, confirming a requirement for αvβ3 in promoting CD4 T-T interactions and associated type-2 cytokine expression *in vivo*.

Taken together, our results highlight roles for αvβ3 in T cell activation and differentiation: mediating early T_H_ cell clustering that promotes T cell activation and proliferation via an IL-2, STAT5, CD25 feedback loop; and a selective and critical role in T_H_2 cell differentiation through the upregulation of αvβ3 by IL-4 to promote FAK-mTOR signalling and IL-13/IL-5 production.

## Discussion

Since their discovery by Mossman and Coffman over three decades ago, the biology of T_H_2 cells has been studied extensively and a myriad of proteins have been implicated in T_H_2 cell differentiation, including transcription factors, signalling molecules and secreted factors ^1, 2^. Complementing more targeted approaches in the past, recent advances in high-throughput techniques including CRISPR-Cas9 mediated genetic screens have allowed biological processes to be interrogated in an unbiased manner ^30^. Here, a whole genome CRISPR-Cas9 mediated knockout screen identified existing and previously unappreciated regulators of T_H_2 cell differentiation. These included the metabolic regulator Acly (ATP citrate lyase, which has been implicated previously in T_H_1 ^31^, but not T_H_2, cell differentiation), that metabolises citrate to produce acetyl-coenzyme A (acetyl-CoA) for histone acetylation in response to cell activation and differentiation ^32^. Such epigenetic remodelling also correlates with our identification of bromodomain-containing proteins Brd2 and Brd4 that bind to acetylated lysine molecules, and can be inhibited by the pan BET-bromodomain inhibitor iBET151 to restrict type 1 and type 2 cytokine expression by T cells and innate lymphocytes and suppress inflammation ^33, 34^. Interestingly, our results indicate that Brd2 and Brd4 play restricted and non-redundant roles in T_H_2 cell differentiation. Our screen also identified the lysine methyltransferases *(Kmt2c* and *Kmt2d)*. Kmt2 proteins have not been studied in the context of T_H_ cell differentiation. However, G9a (encoded by *Kmt1c)* di-methylates H3K9 and T_H_ cells from G9a-deficient mice display increased IL-17A expression with a concomitant decrease in type-2 cytokines ^35^. Whether Kmt2c and Kmt2d perform similar TH-specific functions remains to be determined.

Unexpectedly, we also identified core-binding factor beta (Cbfβ) as functionally important in IL-13 expression and T_H_2 cell differentiation. Cbfβ forms heterodimers with the DNA-binding Runx family of transcription factors. However, none of the Runx proteins were identified in our screens as being functionally required for the T_H_2 differentiation. Indeed, Runx1, Runx3 and their common binding subunit Cbfβ have been characterised as negative regulators of T_H_2 cell differentiation ^36, 37, 38, 39^. This dichotomy may be explained by the recent identification of a role for Cbfβ in promoting mRNA translation in combination with Hnrnpk and eIF4b ^40^. Notably, the authors identified *Gata3* transcripts as targets of this complex. This raises the possibility that targeting Cbfβ results in reduced Gata3 translation and impaired T_H_2 differentiation. This would represent a novel mechanism for regulating T_H_2 development and warrants further investigation.

Strikingly, the screen also identified the αvβ3 integrin cell adhesion and signalling module, which has not been associated previously with T_H_2 cell differentiation. Integrin binding is subject to multiple layers of regulation including changes in expression levels and inside-out signalling of existing integrin molecules. Inside-out activation of integrin can confer rapid adhesion competency independent of transcription or translation, a mode of regulation that is important for homotypic and heterotypic cell adhesion and migration through tissues *in vivo* ^14^. In addition to the integrin subunits the integrin-associated molecules Talin, RIAM and Kindlin3 were also isolated as being required for T_H_2 cell differentiation and IL-13 expression. By contrast, we did not identify the prototypical lymphocyte integrin LFA-1 composed of CD11a (αL, *Itgal)* or CD18 (β2, *Itgb2*) in our screens. This was surprising as LFA-1 has been well-characterised in mediating inside-out signalling following TCR ligation as part of the T cell synapse, especially in T_H_1 responses ^5, 6, 7^, and suggested that the αvβ3 integrin dimer may play a similar role in T_H_2 cell differentiation. Indeed, we found that αvβ3 was induced on T_H_2 cells, but not on T_H_1 cells, leading to profound preferential expression of αvβ3 relative to LFA-1 on T_H_2 versus T_H_1 cells. We further demonstrated that αv and β3 expression is regulated by the IL-4-induced T_H_2 master regulator Gata3 ^19^. This differential expression provides a mechanism by which αvβ3-mediated cell clustering and signalling promotes T_H_2 cell-specific differentiation. Previous reports have highlighted roles for T cell-expressed αvβ3 in promoting migration of T_H_1 cells into inflamed tissues ^41^ and the accumulation of CD4^+^ follicular helper T cells in germinal centres and downstream B cell responses ^42^. Furthermore, αvβ3 upregulation in T_H_2 cells has also been reported to increase T_H_2 cell motility in the absence of chemokine cues, as compared to T_H_1 cells, a process that has been suggested to enhance T_H_2 cell interaction with innate immune cells and stromal cells during inflammation or repair ^43^. Our results now indicate that αvβ3 is also directly playing a key role in establishing and potentiating T_H_2 polarization and cytokine expression.

We observed that during the primary phase of pan-TCR-induced T cell activation αvβ3 is required for homotypic T-T cell conjugate formation and proliferation, showing that this can contribute to the initial broad spectrum T cell response to antigen challenge prior to functional cytokine specialisation. We determined that although calcium-mediated signals downstream of the T cell receptor were not impacted by the absence of αvβ3, IL-2-mediated STAT5 activation was impaired, mirroring the effects reported for LFA-1^8^. Indeed, since the activation phenotype was not observed in our *in vivo* studies, this suggest that additional molecules such as LFA1 can play redundant roles during the initiation of pan-T cell stimulation by antigen. Regulation of quorum sensing among T cells has been highlighted as an important mechanism modulating T cell activation and immune responses, and our results suggest that in T_H_2 lymphocytes αvβ3 may be playing a homologous role to ICAM1-LFA1 interactions in CD8 and T_H_1 cells ^44^.

By contrast, the contribution of IL-4-induced αvβ3 upregulation to T_H_2 cell differentiation and cytokine bias was robustly reproduced *in vivo* using a conditional mouse model of αv-deficiency in T cells, and could also be separated experimentally *in vitro* from the early activation phenotype. Thus higher αvβ3 expression promotes ligand interaction to establish a T_H_2-potentiating microenvironment. Integrins display highly promiscuous ligand binding interactions comprising of intercellular and matrix-associated cognate ligands. Indeed, our gene expression analysis indicated at least 15 candidate ligands for αvβ3 expressed by T_H_ cell subsets, suggesting that one ligand is unlikely to underlie the observed αvβ3-mediated phenotype. However, Thy-1 (CD9O) was an attractive candidate, being highly expressed by T cells, and associated with T cell function ^28^. Blocking Thy-1 reduced type-2 cytokine expression and T cell clustering, and exogenous stimulation with recombinant Thy-1 promoted IL-13 expression by T_H_2 cells in an αvβ3 integrin-dependent manner, demonstrating that Thy-1 can act as a physiological ligand for αvβ3 on T cells.

Downstream of extracellular ligand interactions integrins are known to engage with a signalling platform that commonly includes FAK and Pyk2. We confirmed the role of FAK or Pyk2 in mediating intracellular signalling downstream of αvβ3. Interestingly, as shown previously, FAK can activate the PI3K/mTOR signalling axis ^45, 46^. This is consistent with our CRISPR screens results which highlighted roles for PI3K and mTOR, and our identification of a defect in mTOR-regulated gene expression in αvβ3-deficient T_H_2 cells. Of the mTOR-regulated genes identified in our transcriptomic analyses, *Egr1* and *Egfr* are preferentially expressed by T_H_2 cells. Egr1 has been reported to regulate T cell type-2 cytokine production^47^ and mast cell IL-13 production^48^. EGFR expression on T cells is required for IL-13 expression in vivo^49^ and amphiregulin-mediated IL-9 expression^50^. Thus, our results suggest a pathway by which ligand-mediated intercellular interactions by αvβ3-Thyl (or other ligands) activate mTOR signalling to support cytokine production during type-2 immune reactions.

We employed two experimental models of type 2 immunity to confirm the importance of αvβ3 *in vivo:* OVA-induced lung allergy and papain-induced lung inflammation. In the context of both allergic and antigen-specific immunity, production of IL-5 and IL-13, but not the T_FH_- associated IL-4, were reduced in αvβ3-deficient mice. This deficiency was selective to T_H_2 responses because various aspects of T_H_1 immunity were either unaffected or even elevated. Indeed, our in vitro gene expression analysis suggested that in the absence of αvβ3, Runx3 (an important transcription factor contributing to T_H_1 and suppressor of T_H_2 cell differentiation) is aberrantly upregulated suggesting that such pathways may impact T_H_1 polarisation. Furthermore, as has been shown in many instances, the dysregulation of T_H_1/2 cytokine environments are often reciprocal as observed in our *in vivo* experiments. Additional studies would be necessary to further untangle the potentially complex underlying mechanisms contributing to T_H_1 polarisation. Notably, we found that human T_H_2 cells also differentially express αvβ3, and that this expression is correlated with the co-expression of the asthma-associated cytokine IL-13. Furthermore, nucleotide polymorphisms in human *ITGB3* have been associated with asthma and allergies ^51, 52^, and the ITGAV locus has also recently been linked to asthma following analysis of data from the UK Biobank and the Trans-National Asthma Genetic Consortium ^53^. Although our studies have focused on the T cell derived ligand Thy-1, other ligand possibilities exist on T cells, as well as non-T cell associated matricellular proteins, for example periostin which is a diagnostic marker for allergic asthma ^54^. Our results suggest that the αvβ3-mediated T-T cell interactions may also contribute to the roles of αvβ3 in human disease, and that human ITGB3 and ITGAV may represent potential therapeutic targets in asthma.

**Extended Fig. 6:**
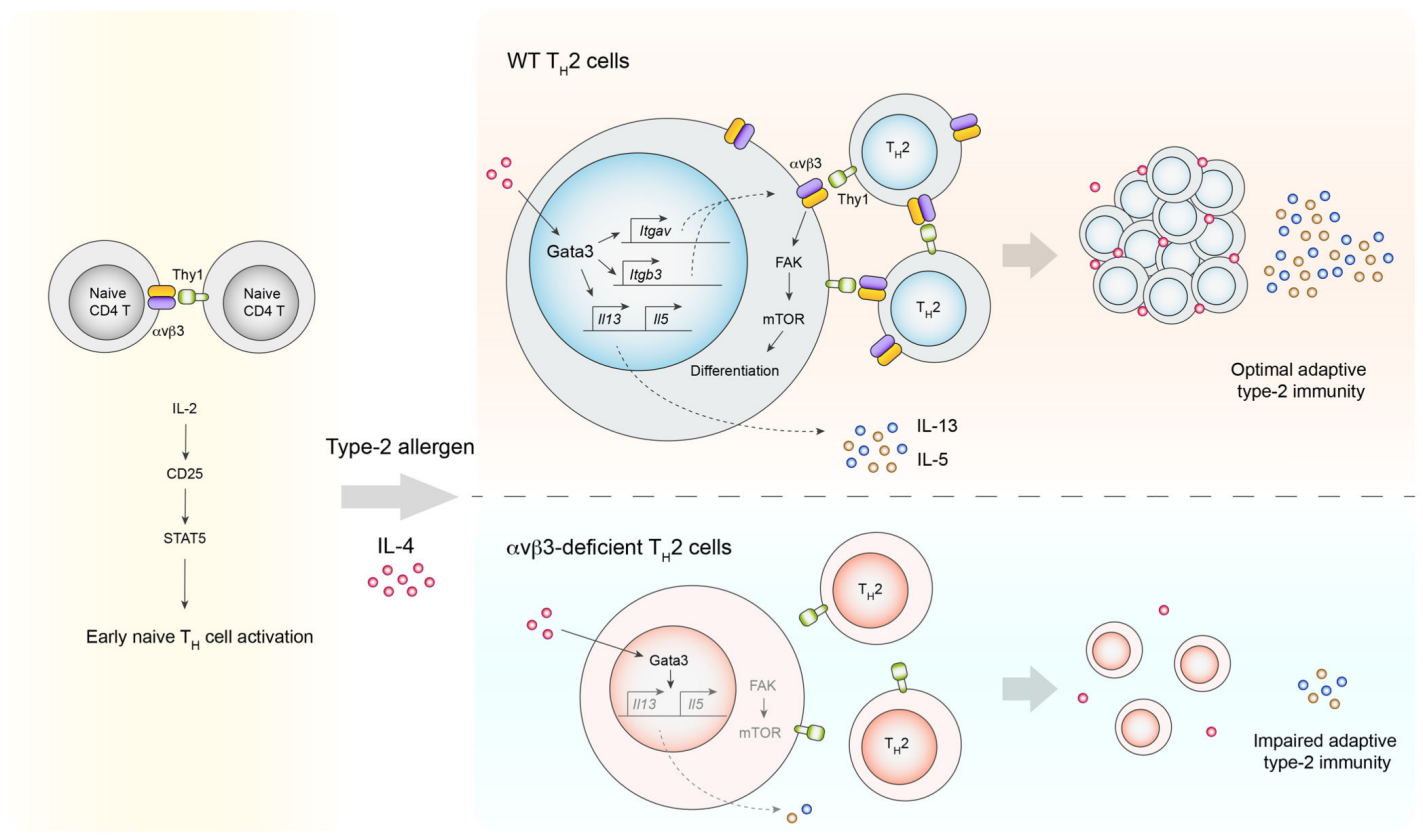
Proposed model for αvβ3-mediated potentiation of T_H_2 cell responses. Naïve CD4^+^ T cells express low levels of integrin αvβ3 which contribute to T cell activation and signalling via the IL-2/CD25/STAT5 axis. IL-4-mediated Gata3 induction upregulates αvβ3 during T_H_2 cell differentiation, permitting intercellular interactions among T_H_2 cells via αvβ3-Thy1 binding. Such interactions enhance mTOR signalling and support optimal T_H_2 responses *in vivo*.

In summary, we propose that αvβ3 is a contributory factor in the early activation of antigen-driven T cell expansion and that its selective upregulation by the IL-4/Gata3 axis is essential for the promotion of intercellular receptor-ligand binding, enhancing mTOR signalling to promote differentiation during the establishment of a specialised cellular microenvironment for the production of T_H_2 cell cytokines (Extended Fig. 6). These type-2 cytokine factories would be expected to increase the local concentration of IL-2 and IL-4 in T-T cell conjugates to increase the likelihood that neighbouring T cells receive polarising IL-4 and co-stimulatory signals to help propagate and amplify T_H_2 responses.

## Methods

### Mice

*Rosa26*^Cas9EGFP^ (JAX 026179)^1^, *Il13*^tdTom 2^, *ItgaV*^fl^ (JAX 032297)^3^, *Itgb3*^fl^ (JAX 028232)^4^, *Cd4*^Cre^ (Taconic, model #4196), *Cd4*^CreERT2^ (JAX 022356)^5^ were on the C57BL/6 background. C57BL/6 controls were bred in-house. All mice were maintained in the Medical Research Council ARES animal facility under specific pathogen-free conditions, at 19-23°C, 45-65% humidity, with a 12-h light-dark cycle. In individual experiments, mice were matched for age, sex and background strain and all experiments undertaken in this study were done so with the approval of the LMB Animal Welfare and Ethical Review Body (AWERB) and of the UK Home Office.

### *In vivo* stimulation

In the OVA/Alum model, mice were injected via the intraperitoneal route with endotoxin-free ovalbumin (20 μg, InvivoGen #vac-pova-100) emulsified in 100 μL Imject Alum Adjuvant (Thermo Scientific #77161) on days 0 and 12, followed by inhalation of nebulised 1% ovalbumin solution on days 19, 21, and 22. Mice were sacrificed for analysis on day 22. In the 2W1S-antigen stimulation models, mice were anesthetized by isoflurane inhalation followed by the intranasal injection of 2W1S peptide (50 μg, Designer Bioscience) in combination with papain (7.5 μg for males, 5 μg for females, Sigma-Aldrich #76216) or LPS (2 μg, InvivoGen #tlrl-3pelps) in 40 μl PBS on days 0 and 14. Mice were sacrificed for analysis on day 19.

### Tissue preparation

Cell suspensions from spleen, lymph nodes, and thymus tissue were obtained by passing the tissues through a 70-μm strainer. Lung tissue was predigested with 750 U ml^-1^ collagenase I (Gibco) and 0.3 mg ml^-1^ DNaseI (Sigma-Aldrich) before obtaining a single-cell suspension. Bone marrow was removed from femurs and tibiae by flushing with PBS, 2% FCS or by centrifuging briefly at 6,000*g*. For bone marrow, lung, and spleen cell suspensions, red blood cells were removed by incubating with RBC lysis solution (140 mM NH4Cl, 17 mM Tris, pH 7.2). Lung lymphocytes were further enriched by centrifugation in 30% Percoll at 800*g* (GE Healthcare).

### Flow cytometry

Single-cell suspensions were incubated with fluorochrome- or biotin-conjugated antibodies in the presence of anti-CD16/CD32 (Fc block, clone 2.4G2) as indicated. Antibodies were from BioLegend (CD3e (BV510 or PE-Cy7, 145-2C11, 1:300 dilution), CD4 (BV605 or PE-Cy7, GK1.5, 1:500 dilution) or (BV785, RM-4-5, 1:500 dilution), CD8a (BV421 or BV785, 53-6.7, 1:500 dilution), CD11a (PE-Cy7, M17/4, 1:500 dilution), CD11b (PE-Cy7, M1/70, 1:1000 dilution), CD19 (BV605, 6D5, 1:500 dilution), CD25 (BV510, PC61, 1:300 dilution), CD29 (Alexa Fluor 700, HMβ1-1, 1:500 dilution), CD31 (BV605, 390, 1:500 dilution), CD44 (PerCP/Cy5.5, IM7, 1:500 dilution), CD45 (BV510, 30-F11, 1:500 dilution), CD49d (PE-Cy7, R1-2, 1:500 dilution), CD51 (biotin or PE, RMV-7, 1:250 dilution), CD61 (biotin or FITC, 2C9.G2, 1:500 dilution), CD62L (BV421, MEL-14, 1:500 dilution), CD90.2/Thy1 (Alexa Fluor 700, 30-H12, 1:500 dilution), CD127 (biotin, SB/199, 1:500 dilution), IL-5 (APC, TRFK5, 1:300 dilution) IFN-γ (BV785, XMG1.2, 1:300 dilution), Sca-1 (BV605, D7, 1:500 dilution), Streptavidin (BV421, 1:500 dilution), T-bet (BV421,4B10, 1:150 dilution), TNF-α (PE or Alexa Fluor 700, MP6-XT22, 1:300 dilution)), eBioscience (CD3e (Alexa Fluor 700, 17A2, 1:300 dilution), CD4 (FITC, GK1.5, 1:500 dilution), CD8a (FITC or PE-Cy7, 53-6.7, 1:500 dilution), CD11c (Alexa Fluor 700 or PE-Cy7, N418, 1:500 dilution), CD19 (PerCP-Cy5.5 or PE-Cy7, eBio1D3, 1:500 dilution), CD44 (FITC or APC, IM7, 1:500 dilution), CD45 (FITC, 30-F11, 1:500 dilution), CD69 (PerCP-Cy5.5, H1.2F3, 1:500 dilution), FceR1 (PE-Cy7, MAR-1, 1:500 dilution), Flt3 (PerCP-eFluor710, A2F10, 1:200), Foxp3 (PE-Cy7, FJK-16s, 1:300 dilution), Gata-3 (eFluor 660, TWAJ, 1:300 dilution), GR-1/Ly-6G/C (PE-Cy7, RB6-8C5, 1:500 dilution), IL-13 (PE or PE-Cy7, eBio13A, 1:300 dilution), MHCII (eFluor450, M5/114.15.2, 1:1000 dilution), NK1.1 (PE-Cy7, PK136, 1:500 dilution), NKp46 (PerCP-eFluo710, 29A1.4, 1:300 dilution), TER-119 (PE-Cy7, TER-119, 1:500 dilution)), BD Biosciences (NK1.1 (BUV395, PK136, 1:300 dilution), Runx3 (PE, R3-5G4, 1:100 dilution), SiglecF (Alexa Fluor 647, E50-2440, 1:500 dilution), Stat5(pY694) (Alexa Fluor 647, 47/Stat5(pY694), 1:25 dilution)) and the NIH Tetramer Facility (2W1S-tetramer, PE, 1:500 dilution). ‘Lineage’ staining included antibodies specific for CD3, CD4, CD8, CD11b, CD11c, CD19, FceRI, GR-1, NK1.1 and TER-119. Human antibodies were from Biolegend (PE, CD51, 1:200 dilution) and eBioscience (IL-13 (PE-Cy7, 85BRD, 1:300 dilution), IFN-γ (Alexa Fluor 700, 4S.B3, 1:300 dilution). All samples were co-stained with a cell viability dye (Fixable Dye eFluor 780, Invitrogen), and analysis was performed on an LSRFortessa system (BD Biosciences) with FACSDiva software (version 6.2, BD Biosciences). For cell sorting, an iCyt Synergy system (70-μm nozzle, Sony Biotechnology) was used. Intracellular cytokine staining was performed using BD Cytofix/Cytoperm Plus reagents (BD Biosciences) following pre-culture with RPMI, supplemented with 50 ng ml^-1^ phorbol 12-myristate 13-acetate (PMA), 500 ng ml^-1^ ionomycin and Protein T ransport Inhibitor Cocktail (eBioscience), for 4 h at 37°C. Intracellular TF staining was performed using Foxp3 Staining kit reagents (eBioscience). Intracellular phospho-Stat5 staining was performed by fixation with 2% PFA for 15 min, overnight permeabilization with 90% methanol at -20°C, followed by incubation with fluorochrome antibodies diluted in 2% BSA PBS. For calcium flux analysis, splenocytes were stained for surface markers, incubated with indo-1 for 30 mins at 37°C, then 1mM CaCl for 30 mins at 37°C. Baseline, CD3e antibody (145-2C11)- and ionomycin-induced calcium flux were measured as Indo-1(Violet)/Indo-1(Blue) ratio. Data were analyzed with FlowJo software (version 10).

### sgRNA cloning into retroviral expression vector

MSCV-pU6-(BbsI)-CcdB-(BbsI)-Pgk-Puro-T2A-BFP was a gift from Ralf Kuehn (Addgene plasmid # 86457; http://n2t.net/addgene:86457; RRID:Addgene_86457) ^6^. Mouse Brie CRISPR knockout pooled library was a gift from David Root and John Doench (Addgene #73633) ^7^. Custom sgRNA libraries were synthesised by Twist Bioscience. sgRNA libraries were cloned into the retroviral vector by Gibson assembly. sgRNA library representation was verified by next generation sequencing to contain > 90% perfectly matching sgRNAs, < 0.5% undetected sgRNAs and a skew ratio of less than 10. sgRNA oligo pairs were purchased from Sigma-Aldrich. Individual CRISPR sequences were inserted into the retroviral vector by ligation (NEB T4 DNA ligase). Sequences of individual sgRNA-expressing constructs were confirmed by Sanger sequencing.

### Generation of retroviral Gata3 overexpression construct

pMIGII-Gata3 was generated by inserting Gata3 cDNA into pMIGII (Addgene, 52107) that was linearized with EcoRI and BamHI, using Gibson assembly. Retroviral production and transduction were performed as described below.

### T_H_2 cell culture for CRISPR screening

Splenic naïve CD4^+^ T cells were sorted as Live CD4^+^ CD44^lo^ CD62L^hi^ CD25^-^ cells. Cells were maintained in RPMI1640, 10% FCS with penicillin-streptomycin and 2-mercaptoethanol. Naïve CD4^+^ T cells were isolated from *Rosa26*^Cas9EGFP^ x *Il13*^tdTom^ mice and cultured on anti-CD3 coated plates (2B Scientific, 145-2C11, 5 μg ml^-1^, 37°C, 1 h), supplemented with anti-CD28 (2B Scientific, 37.51,2 μg ml^-1^) and IL-2 (10 ng ml^-1^) for 24 hr. Cells were collected and mixed with retroviruses and spinoculated on retronectin-coated plates (Takara, 4 μg cm^-2^, non-TC-treated plate) at 37°C for 1 h. Cells were incubated further for 3 h at 37°C before transfer to fresh TC-treated plates until day 6. Fresh media containing 10 ng ml^-1^ IL-2 was supplemented at day 3. On day 6, cells were transferred to anti-CD3 coated plates and cultured in the presence of anti-CD28 (2 μg ml^-1^), IL-2 (10 ng ml^-1^), IL-4 (10 ng ml^-1^) and anti-IFNγ neutralising antibody (2B Scientific, 1 μg ml^-1^). After 3 days of differentiation, GFP^+^ BFP^+^ cells were sorted into IL13Tom^+^ and IL13Tom^-^ populations.

### Retroviral production

Platinum-E retroviral packaging cells (Cell biolabs, #RV-101) were maintained in DMEM, 10% FCS with penicillin-streptomycin, supplemented with puromycin (1 μg ml^-1^) and blasticidin (10 μg ml^-1^). On the day before transfection, 3 million cells were seeded in a 100 mm culture dish in 10 ml of media without antibiotics. Cells were transfected at 70% confluency using Fugene HD Transfection Reagent (Promega). For each 100 mm culture dish, 950 μl OPTI-MEM (GIBCO) was mixed with 11 μg pCl-Eco, 22 μg library plasmid and 99 μl Fugene HD. The transfection mixture was incubated for 10 min at room temperature prior to addition. At 18 h post-transfection, the media was replaced with 10 ml fresh media, and viral supernatant was harvested at 48 and 72 h post-transfection. Cells were removed by filtering through a 0.45 μm syringe filter.

### Genomic extraction and sequencing library preparation

Genomic DNA from sorted cells were extracted using the QIAGEN DNeasy Blood & Tissue Kits following the manufacturer’s protocol, with the exception of DNA elution in water instead of buffer AE. sgRNA-insert was first PCR-amplified using Herculase II Fusion DNA polymerase (Agilent) with primers (Forward) AATGGACTATCATATGCTTACCGTAACTTGAAAGTATTTCG and (Reverse) CTTTAGTTTGTATGTCTGTTGCTATTATGTCTACTATTCTTTCC, using up to 2 μg genomic DNA per 50 μl reaction. Equal volumes from each reaction were pooled and used for a further PCR amplification step to attach Illumina sequencing adaptors and Illumina P7 barcodes, using Herculase II Fusion DNA polymerase. The 330 bp library was gel purified and quantified using KAPA library quantification kit (Roche). Libraries were pooled and sequenced with a HiSeq 4000 at the CRUK Cambridge NGS facility.

### Analysis of CRISPR screen results

20 nt sgRNA sequences were trimmed from backbone sequences using Cutadapt (version 1.4.1) (5’ GACGAAACACCG, 3’ GTTTTAGAGCTA). sgRNA sequences were aligned to reference sgRNA libraries using Bowtie2 (version 1.2.3). sgRNAs with counts less than 20 (genome-wide screens) or 50 (all other screens) in either of the populations were excluded from the analysis. The stat.wilcox function from the caRpools package (version 0.83) was applied to each screen separately using R (v4.1.1). The function was modified to return the non-adjusted p-values. The stat.wilcox function collapses the sgRNAs to genes returning an enrichment score and a p-value for each gene. NT sgRNAs were used as a reference population. To combine data from screen replicates, the mean of enrichment score for each gene was calculated, and Fisher’s method was used to combine the p-values.

### *In vitro* mouse T_H_ cell culture

In differentiation assays, 250,000 splenic naïve CD4^+^ T cells per well were cultured on anti-CD3 coated plates (5 μg ml^-1^), supplemented with anti-CD28 (2 μg ml^-1^) and IL-2 (10 ng ml^-1^) in neutral condition (Th0). The following cytokines and neutralising antibodies were additionally supplemented in different T_H_ conditions. T_H_1: IL-12 (10 ng ml^-1^) and anti-IL-4 neutralising antibody (1 μg ml^-1^). T_H_2: IL-4 (10 ng ml^-1^) and anti-IFNγ neutralising antibody (BioLegend, 11B11, 1 μg ml^-1^). T_re_g: TGFb (5 ng ml^-1^), anti-IL-4 neutralising antibody (1 μg ml^-1^) and anti-IFN-γ neutralising antibody (1 μg ml^-1^). T_H_17: IL-6 (20 ng ml^-1^), IL-23 (10 ng ml^-1^), IL-1b (10 ng ml^-1^), TGFb (2 ng ml^-1^), anti-IL-4 neutralising antibody (1 μg ml^-1^), anti-IFN-γ neutralising antibody (1 μg ml^-1^) and anti-IL-2 neutralising antibody (2B Scientific, JES6-1A12, 1 μg ml^-1^). IL-2 was not supplemented in T_H_17 condition. Cells were passaged on day 2 or day 3, then analysed by flow cytometry on day 5. For clustering analysis, 400,000 cells per well were cultured on anti-CD3 coated plates in the presence of anti-CD28 (2 μg ml^-1^). Cell clusters were imaged at 20 h. In Celltrace dilution experiments, cells were incubated with CellTrace Violet (C34557) according to the manufacturer’s instructions. Where appropriate, neutralising antibodies, recombinant proteins and chemical inhibitors were supplemented at the indicated concentrations and time points. 4-Hydroxytamoxifen (Merck, SML1666) was used at 2.5 μM. LEAF-/Ultra-LEAF purified antibodies were from BioLegend (CD51 (RMV-7, 10 μg ml^-1^), CD61 (2C9.G2, 10 μg ml^-1^), CD90 (30-H12, 10 μg ml^-1^)). Cilengitide was from Generon (HY-16141, 0.5 μg ml^-1^). PF-271 (PZ0387, 1 μM) and PP242 (475988, 0.5 μM) were from Merck. ZINC40099027 (Zn27) was from Generon (AOB33456-1, 0.1 μM) and was included from d0 to d5 for cytokine analysis or at d5 for 30 minutes for phospho-S6 analysis. For Thy-1-Fc stimulation assays, recombinant Thy-1-Fc (BioTechne, 7335-CD-050) or Fc (InVivoMab, BE0097) was conjugated to M-450 Epoxy Dynabeads (Invitrogen, 14011) according to the manufacturer’s instructions. Conjugated beads were cultured with cells at a 10:1 ratio to minimise endogenous cell-cell contact and promote bead-cell contacts.

### Human T_H_ cell culture

Blood was obtained from volunteers (patients with asthma and healthy controls). UK HRA approval was granted following Research Ethics Committee (North West-Liverpool Central) review and written consent obtained from volunteers. Peripheral blood naïve CD4^+^ T cells were purified using the naive CD4+ T cell Isolation Kit II (Miltenyi Biotec, 130-094-131) according to the manufacturer’s instructions. 250,000 splenic naïve CD4^+^ T cells per well were cultured on anti-CD3 coated plates (clone OKT3, 5 μg ml^-1^, BioLegend 317325) supplemented with anti-CD28 (clone CD28.2, 2 μg ml^-1^, BioLegend 302933) and IL-2 (10 ng ml^-1^, R&D 204-IL-010), and additionally IL-4 (12.5 ng ml^-1^, R&D 202-IL-010) and anti-IFNγ neutralising antibody (clone B27, 1 μg ml^-1^, BioLegend, 506531) in T_H_2 conditions, or IL-12 (2.5 ng ml^-1^, R&D 219-IL-005) and anti-IL-4 neutralising antibody (clone MP425D2, 1 μg ml^-1^, BioLegend, 500837) in T_H_1 conditions. Cells were cultured for 4 weeks with a weekly stimulation (4 days) and rest (3 days) schedule.

### Cell clustering analysis

Image analysis was performed using ImageJ (Fiji). Following background subtraction, conversion into 8-bit image and threshold adjustment, clusters and percentage area covered by particles were quantified. For flow cytometric analysis of doublet formation, cells were stained with surface markers as described above, followed by incubation in complete RPMI for 15 minutes at 37°C to enable T-T conjugate formation. Conjugates were fixed with 2% PFA for 15 minutes at room temperature prior to flow cytometric analysis.

### RNA-sequencing

Cells were sorted by flow cytometry into PBS, 50% FCS, and RNA was extracted using the RNeasy Plus Micro kit (Qiagen). After assessment using a Bioanalyzer (Agilent), RNA was processed for RNA-seq using an Ovation RNA-seq System V2 (Nugen), fragmented using the Covaris M220 ultrasonicator and bar-coded using Ovation Ultralow Library Systems (Nugen). Samples were sequenced using an Illumina HiSeq 4000, by running a single-read 50-bp protocol (Cancer Research UK Cambridge Institute). Sequence data were trimmed to remove adaptors and sequences with a quality score below 30 using Trim Galore (version 0.50, Babraham Bioinformatics) and then aligned to the mouse genome (GRCm38) using STAR (version 2.6.0a), and differential expression was calculated using DESeq2 (version 1.18.1) ^8^.

### RT-qPCR

RNA was purified using QIAGEN RNeasy Mini Kit. cDNA synthesis was performed using SuperScript IV Reverse Transcriptase (Invitrogen). Diluted cDNA (1:20) was used as template for SYBR green or Taqman qPCR assays.

### Immunoprecipitation

T_H_2 cells were lysed in lysis buffer (30 mM Tris pH 7.4, 120 mM NaCl, 2 mM KCL, 1% Triton-X-100 and 2 mM EDTA), supplemented with 1x cOmplete protease inhibitor (Roche) and PMSF (Sigma Aldrich). Lysates were centrifuged at >16,000 *g* at 4°C for 30 min, and the supernatant was collected. Protein concentration was quantified using the Pierce 660nm protein assay reagent (ThermoFisher, #22660). Lysates were incubated with antibodies (2 μg antibody per 100 μg protein) overnight at 4°C on a rotator. CD51 antibody was from Abcam (#ab179475) and CD61 antibody was from BioLegend (#104302). Immunocomplexes were precipitated with protein A/G dynabeads (Thermo Scientific #88802), washed three times with lysis buffer and once with TE buffer (10 mM Tris and 0.1 mM EDTA, pH 8). For western blot analysis, cell lysates or immunocomplexes were denatured by boiling at 95°C for 5 min in 1X NuPage LDS sample buffer (#NP0008) supplemented with 1% 2-mercaptoethanol. Proteins were resolved with Novex Tris-Glycine gels and transferred to PVDF membranes. Membranes were sequentially blocked with 5% BSA in PBST, incubated with anti-Thy1 primary antibody (R&D #MAB733) and HRP-conjugated anti-rat secondary antibody (Santa Cruz #sc-2065), and ECL western blotting detection reagent (GE Healthcare #RPN2106).

### Gata3 ChIP-seq using ChIPmentation

Chromatin extracts from *in vitro* T_H_2 cells (1.0 × 10^7^ cells) were prepared using the truChIP Chromatin Shearing kit (Covaris), with 5 min of crosslinking and optimized shearing conditions (peak power, 75; duty factor, 10.0; cycles per burst, 200; duration, 300 s), to obtain fragments of ~500bp. Extracts were exposed to 1% SDS and diluted 10x with dilution buffer (5.5 mM EDTA, 55 mM Tris-HCl, pH 8, 200 mM NaCl, 0.5% NP-40). Chromatin extracts were incubated overnight at 4 °C with 2 μg anti-Gata3. In addition, 25 μl protein A Dynabeads (Thermo Fisher Scientific) per immunoprecipitation were blocked in PBS containing 0.1% BSA (Sigma) by incubating overnight at 4 °C. The next day, beads were added to the chromatin extracts, followed by incubating for 1 h at 4 °C. Beads were collected and washed twice with low-salt buffer (0.1% SDS, 1% Triton X-100, 1 mM EDTA, 10 mM Tris-HCl, pH 8, 140 mM NaCl, 0.1% sodium deoxycholate), twice with high-salt buffer (0.1% SDS, 1% Triton X-100, 1 mM EDTA, 10 mM Tris-HCl, pH 8, 500 mM NaCl, 0.1% sodium deoxycholate), twice with LiCl buffer (10 mM Tris-HCl, pH 8, 1 mM EDTA, 250 mM LiCl, 0.5% NP-40, 0.5% sodium deoxycholate) and once with 10 mM Tris-HCl, pH 8. Chromatin-antibody-bead complexes were then subjected to tagmentation, followed by the elution of DNA, and libraries were amplified and purified as described previously ^9^. Pooled libraries were sequenced using an Illumina HiSeq 4000, running a single-read 50-bp protocol (Cancer Research UK Cambridge Institute). Sequenced reads were aligned to the mouse genome (GRCm38) using Bowtie2 (version 2.3.5.1) with default parameters, and reads that could not be uniquely mapped were removed from further analyses. Aligned reads were visualised using the SeqMonk software (v1.48.0).

### Statistical analysis

Statistical analysis was performed using GraphPad Prism version 9 software.

## Extended Data

## Data Availability

All high-throughput data in this study were deposited at the Gene Expression Omnibus (GEO) under accession number GSE179210. Source data are provided with this paper. Th transcriptomic data were obtained from Th-express (https://th-express.org).
